# SubSol-HIe is an AMPK-dependent hypoxia-responsive subnucleus of the nucleus tractus solitarius that coordinates the hypoxic ventilatory response and protects against apnoea in mice

**DOI:** 10.1007/s00424-024-02957-6

**Published:** 2024-04-18

**Authors:** Sandy MacMillan, David P. Burns, Ken D. O’Halloran, A. Mark Evans

**Affiliations:** 1 Centre for Discovery Brain Sciences, College of Medicine and Veterinary Medicine, Hugh Robson Building, University of Edinburgh, Edinburgh, EH8 9XD UK; 2 Department of Physiology, School of Medicine, College of Medicine & Health, University College Cork, Cork, Ireland

**Keywords:** AMPK, NTS, Catecholaminergic, Hypoxia, Active expiration, Hypoxic ventilatory response, Apnoea

## Abstract

Functional magnetic resonance imaging (fMRI) suggests that the hypoxic ventilatory response is facilitated by the AMP-activated protein kinase (AMPK), not at the carotid bodies, but within a subnucleus (Bregma -7.5 to -7.1 mm) of the nucleus tractus solitarius that exhibits right-sided bilateral asymmetry. Here, we map this subnucleus using cFos expression as a surrogate for neuronal activation and mice in which the genes encoding the AMPK-α1 (*Prkaa1*) and AMPK-α2 (*Prkaa2*) catalytic subunits were deleted in catecholaminergic cells by Cre expression via the tyrosine hydroxylase promoter. Comparative analysis of brainstem sections, relative to controls, revealed that AMPK-α1/α2 deletion inhibited, with right-sided bilateral asymmetry, cFos expression in and thus activation of a neuronal cluster that partially spanned three interconnected anatomical nuclei adjacent to the area postrema: SolDL (Bregma -7.44 mm to -7.48 mm), SolDM (Bregma -7.44 mm to -7.48 mm) and SubP (Bregma -7.48 mm to -7.56 mm). This approximates the volume identified by fMRI. Moreover, these nuclei are known to be in receipt of carotid body afferent inputs, and catecholaminergic neurons of SubP and SolDL innervate aspects of the ventrolateral medulla responsible for respiratory rhythmogenesis. Accordingly, AMPK-α1/α2 deletion attenuated hypoxia-evoked increases in minute ventilation (normalised to metabolism), reductions in expiration time, and increases sigh frequency, but increased apnoea frequency during hypoxia. The metabolic response to hypoxia in AMPK-α1/α2 knockout mice and the brainstem and spinal cord catecholamine levels were equivalent to controls. We conclude that within the brainstem an AMPK-dependent, hypoxia-responsive subnucleus partially spans SubP, SolDM and SolDL, namely SubSol-HIe, and is critical to coordination of active expiration, the hypoxic ventilatory response and defence against apnoea.

**Supplementary information:**

The online version contains supplementary material available at 10.1007/s00424-024-02957-6.

## Introduction

When oxygen availability falls, the hypoxic ventilatory response (HVR) delivers compensatory increases in ventilatory drive. The HVR is initiated by increases in afferent fibre discharge from the carotid bodies, the primary peripheral arterial chemoreceptors of mammals, that in turn elicits increases in ventilatory drive via the respiratory central pattern generators (rCPG) in the brainstem [41, 49, 85]. Our previous studies revealed that the HVR is facilitated by a cellular energy sensor, the AMP-activated protein kinase (AMPK), within catecholaminergic neurons of the nucleus tractus solitarius (NTS) that receive carotid body afferent input responses [59], rather than at the level of the carotid bodies [58, 59]. Therefore, the role of AMPK extends beyond cellular metabolic homeostasis [81] to breathing and thus oxygen [21, 23] and energy (ATP) supply to the body as a whole [22].

AMPK maintains cellular energy homeostasis in a cell-autonomous manner, through the action of at least twelve possible isoforms formed by heterotrimeric association of the two α catalytic, two β, and three γ regulatory subunits [81]. Importantly, each may hold different sensitivities to activation by increases in cellular AMP and ADP and differ in their capacity to directly phosphorylate and thus regulate downstream targets^6^. AMPK is coupled to mitochondrial oxidative phosphorylation and thus oxygen supply by two discrete but cooperative pathways. Binding of AMP to the AMPK-γ subunit increases activity tenfold by allosteric action, while AMP or ADP binding delivers increases in LKB1-dependent phosphorylation and reductions in dephosphorylation of Thr172 on the α subunit that confer 100-fold further activation [34]. All of these effects are inhibited by ATP. There are also three AMP- and ADP-independent pathways to AMPK activation: long chain fatty acyl CoA regulation through the allosteric drug and metabolite (ADaM) site [73, 81]; glucose modulation through a fructose-1,6-bisphosphate (FBP) sensing mechanism that may involve the aldolase-v-ATPase-Ragulator complex on lysosomes [51, 94]; and calcium-dependent activation through calcium-calmodulin activated kinase kinase 2 (CaMKK2) [90]. Of these, it is the canonical energy stress and thus the LKB1-AMPK signalling pathway that facilitates the HVR [58].

Classically, once activated, AMPK maintains energy homeostasis in a cell autonomous manner, by activating catabolic and inhibiting anabolic processes [81]. However, AMPK may also regulate cell- and system-specific function [20] by phosphorylating and regulating non-canonical targets including ion channels [5, 13, 40, 46, 63, 67, 75], enzymes for transmitter biosynthesis [95], receptors [2] and transporters [78].

Functional magnetic resonance imaging indicated that AMPK deletion in catecholaminergic neurons preferentially attenuates activation of a subnucleus of the NTS with right-sided bilateral asymmetry [59]; note, bilateral asymmetry or symmetry describes units separated by a midline axis, across which symmetry or asymmetry is observed. Importantly, carotid body afferent inputs terminate within this region of the NTS [25], which lies adjacent to the caudal most aspect of the area postrema (AP). Here, within the dorsal vagal complex [36], afferent input responses are integrated before onward relay of output commands to the ventrolateral medulla (VLM) which facilitate increases in ventilatory drive via the ventral rCPG [3, 39, 93]. Although the precise circuit mechanisms remain to be determined, it is evident that the most prominent chemo-afferent projections of the carotid body terminate within the commissural (SolC) and medial (SolM) subnuclei, while less prominent projections are evident at the interstitial (SolI), intermediate (SolIM), dorsolateral (SolDL) and ventrolateral (SolVL) subnuclei. In addition, a few terminals project to the AP and the dorsal motor nucleus of the vagus (10N) [25]. By contrast, vagal afferents arising from the pulmonary stretch receptors terminate within SolIM, SolI, the ventral subnucleus (SolV) and SolVL [48], while densely projecting baroreceptor afferent terminals project to SolM at the caudal most aspect of the AP, where they coincide with afferent innervation from peripheral chemoreceptors [61]. These findings are significant because conditional deletion of AMPK in catecholaminergic neurons attenuates the HVR without dramatically altering systemic cardiovascular responses to hypoxia [66].

It is therefore important that we gain a greater understanding of the site and mechanism of AMPK action. This is especially so given that mice with AMPK deletion targeted to catecholaminergic neurons exhibit a neonate-like HVR during poikilocapnic hypoxia, comprising decreased ventilation and apnoea with preserved hypercapnic ventilatory responses [59], while exposure to hypoxia under anaesthesia triggers irreversible ventilatory arrest [59]. In short, these mice exhibit face validity that is consistent with symptoms in patients suffering from central apnoea of prematurity in neonates [29, 64, 72] and central sleep apnoea in adults, which is allied with susceptibility to respiratory arrest under anaesthesia [69].

We show here that AMPK deletion selectively attenuates, with right-sided bilateral asymmetry, activation during hypoxia of a neuronal cluster that partially spans three anatomical nuclei of the NTS that lie adjacent to the AP, namely SolDL, SolDM and SubP, and at a location that approximates the volume of the AMPK-dependent, hypoxia-responsive region of the NTS (Bregma -7.1 mm to -7.5 mm) identified by fMRI [59]. Accordingly, AMPK deficiency in catecholaminergic neurons blocks hypoxia-evoked decreases in expiration time and increases in sigh frequency and preferentially increased the frequency of apnoeas. These changes result in a profound blunting of the hyperventilatory response to hypoxia (normalised to metabolism).

## Methods

### Mouse models

Experiments were performed in accordance with the regulations of the United Kingdom Animals (Scientific Procedures) Act of 1986. All studies and breeding were approved by the University of Edinburgh and performed under UK Home Office project licenses. Studies performed in Cork were carried out under licence from the Department of Health and Children, Ireland. All mice were C57/Bl6 (wildtype) or bred on C57/Bl6 background and tested between 3 and 12 months of age. Data shown here were from males and/or females. Numbers of mice (≥ 4 per measure) used are indicated for each experiment. Global, dual knockout of the genes encoding AMPK-α1 (*Prkaa1*) and AMPK-α2 (*Prkaa2*) is embryonic lethal. We therefore employed conditional deletion of the genes for the AMPK-α1 and AMPK-α2 subunits, using mice in which the sequence encoding the catalytic site of both α subunits was flanked by loxP sequences [50] (AMPK-α1/α2 Flx). Mice with AMPK deletion in catecholaminergic cells were obtained as described previously by crossing AMPK-α1/α2 Flx mice with mice in which Cre recombinase expression was driven via the tyrosine hydroxylase (TH) promoter [53].

### Genotyping

The presence of wild-type or floxed AMPK-α1 and AMPK-α2 alleles and Cre recombinase [56, 57, 59] was detected by PCR of digested ear clip tissue. Three primers were used to detect TH Cre (forward 5′ CACCCTGACCCAAGCACT 3′; reverse 5′ CTTTCCTTCCTTTATTGAGAT 3′; internal positive control 5′ GATACCTGGCCTGGTCTCG 3′; expected size WT = 290 bp, Cre = 390 bp), and two primers were used for each AMPK catalytic subunit: α1-forward 5′ TATTGCTGCCATTAGGCTAC 3′, α1-reverse: 5′ GACCTGACAGAATAGGATATGCCCAACCTC 3′ (WT = 588 bp, floxed = 682 bp); α2-forward 5′ GCTTAGCACGTTACCCTGGATGG 3′, α2-reverse: 5′ GTTATCAGCCCAACTAATTACAC 3′ (WT = 204 bp, floxed = 250 bp). Also, 10 µl samples were run on 2% agarose gels with 0.01% v/v SYBR®Safe DNA Gel Stain (Invitrogen) in TBE buffer against a 100 bp DNA ladder (GeneRuler™, Fermentas) using a Model 200/2.0 Power Supply (Bio-Rad). Gels were imaged using a Genius Bio Imaging System and GeneSnap software (Syngene).

### Plethysmography

As described previously [56, 57, 59], we used a whole-body unrestrained plethysmograph, incorporating a Halcyon™ low noise pneumatochograph (Buxco Research Systems, UK) coupled to FinePointe acquisition and analysis software (Data Science International, USA). Following acclimation and baseline measurements (awake but quiet, undisturbed periods of breathing) under normoxia (room air), mice were exposed to hypoxia (8% O_2_, with 0.05% CO_2_, balanced with N_2_) for 10 min or 60 min. The FinePointe software automatically calculated the respiratory parameters assessed after application of exclusion criteria due to non-ventilatory artefacts (movement, sniffing, etc.). Data were acquired as 2-s averages of 2 to 4 data points of undisturbed breathing per time point of the HVR. Apnoeas were defined as a period of cessation of breathing that was greater than the average duration, including inter-breath interval, of 2 successive breaths (~ 600 ms) of control mice during normoxia with a detection threshold for inspiration of 0.25 mmHg (SD of the noise).

### Metabolic measurements

Naïve and untrained mice undergoing metabolic measurements (O_2_ consumption (*V*O_2_) and CO_2_ production (*V*CO_2_)) were placed in a whole-body plethysmography chamber as described above (Buxco, USA). Airflow through the chamber was maintained at 1 l/min. Fractional concentrations of O_2_ and CO_2_ were measured in air entering and exiting the plethysmography chamber by an O_2_ and CO_2_ analyser (ADI instruments, Colorado Springs, CO, USA) online data sampling performed at a frequency of 0.1 Hz, with one cumulative value per minute per mouse used for further analysis. To ensure steady-state conditions during hypoxia, metabolism values were taken after 5 min exposure to hypoxia.

Two hypoxic protocols were used for each mouse—a step challenge and a graded hypoxia challenge. The step challenge introduced 8% O_2_ (0.05% CO_2_, balanced in N_2_) immediately following a 5-min baseline period at 21% O_2_ (0.05% CO_2_, balanced in N_2_) and lasted for a total of 10 min. The graded hypoxia challenge involved a 5-min baseline period at 21% O_2_ (0.05% CO_2_, balanced in N_2_), which was then followed by 5 min of decreasing O_2_ concentrations (18% O_2_, 15% O_2_, 12% O_2_, 10% O_2_, 8% O_2_, all in 0.05% CO_2_, balanced in N_2_).

For the hypoxic step challenge, data are shown normalised for body mass (g) after 5 min of hypoxia and presented on a minute-by-minute basis thereafter. For the graded hypoxic challenge, measurements were taken during the final (fifth) minute of each given O_2_ concentration. Ventilatory measurements were acquired during step and graded hypoxia challenges. The ventilatory equivalents for oxygen and carbon dioxide were determined by expressing minute ventilation (*V*E) as a function of oxygen consumption (*V*E/*V*O_2_) and separately as a function of carbon dioxide production (*V*E/*V*CO_2_).

### High-performance liquid chromatography (HPLC) coupled to electrochemical detection for the measurement of brainstem monoamine concentrations

The effect of AMPK deletion in catecholaminergic cells on the brainstem bioamine content was assessed by high performance liquid chromatography (HPLC).

Following completion of all metabolic measurements, experimental mice were returned to their holding cages and allowed to recover and settle for a minimum of 60 min. Mice were culled by exposure to 5% isoflurane in air, and dislocation of the neck and trunk blood was collected immediately and placed on ice. Brainstems and spinal cords were dissected.

Tissues were sonicated (Bandelin Sonopuls HD 2070) in 1 ml of chilled mobile phase, spiked with 2 ng/20 ml of N-methyl 5-HT as internal standard. Brainstem monoamine precursor and metabolite concentrations were measured as previously described [55, 68]. Noradrenaline (NA), serotonin (5-HT), monoamine metabolite and precursor, 5-hydroxyindoleacetic acid (5-HIAA) and L-3,4 dihydroxyphenylalanine (L-DOPA) were identified by their characteristic retention times and concentrations determined by comparison against standard injections run at regular intervals during sample analysis.

### Perfusion, fixation and cryoprotection

Following administration of an overdose of sodium pentobarbital, the chest cavity was exposed, and mice were perfusion-fixed by cardiac puncture with ice-cold heparinised saline containing 4% paraformaldehyde (PFA). The whole brain and cervical spinal cord were extracted and post-fixed overnight in a 4% PFA/15% sucrose post-fix solution at 4 °C. The next day, brains were transferred into a 30% sucrose solution and stored at 4 °C for a minimum of 24 h.

Upon tissue extraction, each specimen was allocated a random three-digit code, and all subsequent processes were carried out blind with respect to the treatment condition until unblinding took place at the point of statistical analysis.

### Sectioning of the brainstem

Brainstems and the cervical part of the spinal cord were separated from the forebrain roughly at the point of the optic chiasm using a sharp blade, and then a further incision was made to the right side of the brainstem in order to differentiate left and right sides during mounting. Each specimen was wrapped in aluminium foil and flash frozen in ground dry ice.

Frozen brainstems were mounted caudal side up onto a Frigomobil freezing microtome (Leica), covered in Cryo-M-Bed embedding compound (Bright) and allowed to freeze again. Sections were cut with a 33° blade at a thickness of 30 μm and collection started as soon as the cerebellum became visible. Seven alternating sections (A’s and B’s), each separated by 60 μm, were collected per vial in 0.1 M phosphate buffer (PB) and later transferred into cryoprotecant (20% v/v glycerol, 30% v/v ethylene glycol, 50% v/v 0.2 M PBS) for storage at -20 °C until used for immunohistochemistry.

### Immunohistochemistry and diaminobenzidine staining

For immunostaining, several vials per animal from the same alternation (either all A’s or all B’s per animal) were used in a free-floating technique. All procedures were carried out at room temperature on a shaker. Sections from each vial were washed four times for 10 min in 0.3% Triton X-100 in 0.1 M phosphate buffered (PBT), followed by a 20-min incubation in 0.3% (v/v) hydrogen peroxide. After another four 10-min washes in 0.1 M PBT, sections were incubated in blocking buffer (3% normal serum against the host species of the secondary antibody) for 30–60 min and then incubated in rabbit anti-cFos primary antibodies (1:50,000; 226 003 Synaptic Systems, Germany) diluted in blocking buffer for an initial 30–60 min at room temperature before being transferred to 4 °C for 36–48 h.

After six 5-min washes in 0.1 M PBT, a biotinylated IgG anti-rabbit secondary antibody (1:500; BA-1100, Vector Laboratories) diluted in blocking buffer was applied for 1 h at room temperature. Sections were rinsed in four 5-min washes with 0.1 M PBT, and antibody‐antigen complexes were visualised by using ABC methods with a vector stain elite kit (Vector Laboratories, UK) with nickel‐intensified diaminobenzidine (Ni‐DAB). Sections were washed in 0.1 M PB twice for 5 min and once for 10 min, and the above protocol repeated with the following changes: The primary antibody used was mouse anti-TH (1:2000; MAB318, Merck Millipore, UK), secondary antibody used a biotinylated IgG anti-mouse (1:500; BA-1100, Vector Laboratories), and antibody‐antigen complexes were visualised by using ABC methods with a Vector stain elite kit intensified with diaminobenzidine only.

Sections were mounted on gelatine-coated glass microscope slides (Fisher) in a caudo-rostral order and air-dried in a sealed container overnight. Dried sections were dehydrated by submerging them in solutions of increasing concentrations of ethanol, (70% v/v, 90% v/v and 95% v/v) for 5 min each, followed by three times 10 min at 100% v/v and finally two 10-min steps in xylene. A glass coverslip was applied using DPX Mountant for histology (Sigma), and all slides were left to dry in a fume hood for 48 h before storage at 4 °C and microscopy.

### Image acquisition

Images of each section were captured using a Leica DMR reflected light microscope and LAS V4.5.0 imaging software (Leica). A 5 × magnification was used to capture an overview the entire section, whereas a 20 × magnification was used to capture images of the dorsal nucleus tractus solitarius (NTS) and area postrema (AP) and the ventrolateral regions that showed TH-positive cell bodies.

For the dorsal NTS regions of interest, three images were stitched into one using the LAS imaging software in order to retain the original image resolution for measurements of surface area.

### Image analysis

Using an online tool, regions of the NTS present in the mouse brain atlas^23^ were selected, and the background made transparent. These “templates” were then overlaid onto each immunohistochemical image using Inkscape version 0.48.2. The template with the best fit for the AP as a landmark was chosen, whereby the size of the template was increased or decreased proportionally to fit the underlying image. Templates were created for each brain map in the mouse brain atlas that included the area postrema, which encompassed Bregmas -7.76 mm, -7.64 mm, -7.56 mm and -7.48 mm. Any sections that could not be clearly allocated to a specific Bregma from the brain atlas—due to smaller distances between sections cut (60 µm) and those illustrated in the atlas (80 µm)—were labelled as -7.70 mm, -7.60 mm, -7.52 mm and -7.44 mm.

The images with the templates were then further analysed using (FIJI Is Just) ImageJ version 2.0.0-rc-43/1.51 s. At this point, all procedures were still carried out blind with respect to treatment condition (normoxia/hypoxia). To measure the surface area for each subnucleus of the NTS, a global scale was set for each image according to the LAS specifications for 20 × magnifications. Using the “ROI” plugin and a free-hand drawing tool, each subnucleus and the AP were traced along the template lines and the surface areas measured. Each of the measurements was then multiplied by 30 to account for the 30-μm thickness of each section. cFos^+^ nuclei within each subnucleus of the NTS and the AP were counted manually using a counter plugin and simultaneously confirmed on the microscope. Data are presented as total cFos^+^ counts per 1000 µm^3^.

### Confocal imaging

To identify neuronal networks in which dual AMPK-α1/α2 deletion had been induced, mice with TH-Cre driven AMPK-α1/α2 deletion were gene deletion that were crossed with mice engineered for Cre-dependent expression of tdTomato (excitation 555 nm, emission 582 nm) from the Rosa26 locus. These mice were deeply anaesthetised by IP injection of 2 g/kg Pentobarbital Sodium (Merial), transcardially perfused with ice-cold heparinised saline and fixed with 4% paraformaldehyde in 0.1 M phosphate buffer (PB; pH 7.4). Brains were extracted, post-fixed and stored in 30% sucrose in 0.1 M PB at 4 °C. And 30-µm sections of the brainstem were cut using a Frigomobil freezing microtome (Leica). Alternate sections were collected together and mounted onto glass slides, briefly air-dried and coverslipped using Vision™ PermaFluor™ Aqueous Mounting Medium (Thermo Fisher).

Brainstem sections were imaged using a Nikon A1R + confocal system and tdTomato autofluorescence detected using an excitation wavelength of 554 nm and emission wavelength of 581 nm. Relevant regions of the caudal brainstem harbouring catecholaminergic neurons were identified using the mouse brain atlas [27].

### Statistical analysis

Once all stained sections from each experimental animal had been counted, unblinding occurred, and statistical comparison was completed using GraphPad Prism 6. Student’s unpaired *t*-test and one-way ANOVA with Tukey post-hoc test was used when comparing across one variable (e.g. genotype), and two-way ANOVA with Sidak post-hoc tests was used when comparing across two variables (e.g. genotype and time). Statistical significance was assumed when *p* ≤ 0.05. To ensure as few mice as possible were used to determine differences by significance test, experiments were conducted and acquired data statistically assessed in stages by the variable criteria sequential stopping rule (SSR). In this way, animal use was minimised, power maximised, and the probability of type I errors kept constant.

## Results

Previously, targeted deletion of AMPK-α1 and AMPK-α2 in brainstem catecholaminergic cells through Cre expression via the tyrosine hydroxylase promoter (TH-AMPK-α1/α2 knockout) was confirmed by single-cell end-point PCR, by viral injection of a Cre-inducible vector carrying a reporter gene [59] and by crossing TH-AMPK-α1/α2 knockout mice with mice engineered for Cre-dependent expression of Rosa (tdTomato) and subsequent analysis by confocal imaging of caudal brainstem areas [57].

### AMPK deletion attenuates cFos expression of a hypoxia-responsive nucleus within the nucleus tractus solitarius

Functional magnetic resonance imaging (fMRI) analysis previously indicated that TH-AMPK-α1/α2 deletion in catecholaminergic neurons resulted in marked attenuation of neuronal activation during hypoxia within a “dorsal active region” (DAR) of the nucleus tractus solitarius (NTS) that exhibited right-sided bilateral asymmetry [59]; note that bilateral asymmetry or symmetry describes units separated by a midline axis, across which symmetry or asymmetry is observed. The overall shape of the DAR indicated that several anatomical subnuclei of the NTS were likely affected by AMPK deficiency. Surprisingly, AMPK deletion and subsequent reductions in neuronal activity within the DAR were associated with attenuation of the hypoxic ventilatory response (HVR) at the peak of the augmenting phase (~ 30 s), roll-off (~ 100 s) and the sustained phase (~ 100 s) [56, 57, 59]**,** but not cardiovascular responses to hypoxia [56]. This is despite the fact that both cardiovascular and respiratory compartments of the ventrolateral medulla (VLM) are in receipt of NTS afferent inputs. This suggests that an AMPK-dependent, hypoxia-responsive subnucleus within the NTS preferentially facilitates respiratory function. Therefore, we sought to identify the precise location of this NTS subnucleus along the neuraxis of TH-AMPK-α1/α2 knockout mice. To this end, we employed immunohistochemical strategies to investigate the expression of cFos as a surrogate for neuronal activation during hypoxia in males only, to insure against any confounding variable due to the oestrus cycle.

**Fig. 1:**
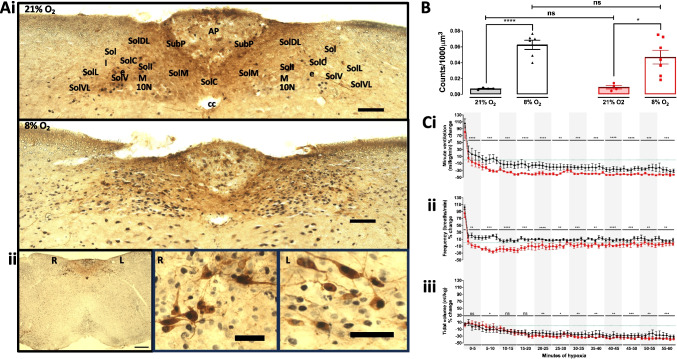
The effect of deleting AMPK-α1/α2 catalytic subunits in catecholaminergic cells on the hypoxic ventilatory response and total dorsal brainstem cFos expression during hypoxia. **Ai** Representative photomicrographs of cFos-positive (black) nuclei in the caudal brainstem of AMPK-α1/α2 floxed mice following 60 min exposures to, *upper panel*, 21% O_2_ and, *lower panel* 8% O_2_. Approximate Bregma -7.56 mm. In this and all subsequent figures, AP = area postrema, cc = central canal, SubP, subpostrema nucleus, SolC = commissural division, SolM = medial nucleus, SolDL = dorsolateral nucleus, SolIM = intermediate nucleus, SolCe, central nucleus, SolI = interstitial nucleus, SolV = ventral nucleus, SolVL = ventrolateral nucleus, SolL = lateral nucleus. Scale bar = 100 μm. **Aii** Exemplar image on the left shows whole brainstem section (right, R; left, L; Scale bar, 300 μM) with the area spanning SolDL, SolDM, SolCe, SolV and SolIM. Middle and righthand panels show high-resolution images (scale bars, 50 μM) of neurons from the same section that are double labelled for cFos + nuclei and tyrosine hydroxylase, proximal to SolDL. **B** Means ± SEM for changes in whole NTS cFos counts (per 1000 μm^3^) in AMPK-α1/α2 floxed (AMPK-α1/α2 Flx, black) and TH-driven AMPK-α1/α2 double knockout mice (TH AMPK-α1/α2 dKO, red) exposed to room air (21% O_2_, shaded bars; controls: *n* = 4, knockouts: *n* = 4) or 60 min of 8% O_2_ (empty bars; controls: *n* = 6, knockouts: *n* = 7). ns = not significant, *p < 0.05, ****p < 0.0001. **C** Means ± SEM for the % change relative to normoxia (green dotted line) in (**i**) minute ventilation, (**ii**) breathing frequency and (**iii**) tidal volume during 60-min exposures to severe hypoxia (8% O_2_) for whole minute averages in AMPK-α1/α2 Flx mice (black, *n* = 5 mice) and TH AMPK-α1/α2 dKO (red, *n* = 4 mice). ns = not significant, *p < 0.05, **p < 0.01, ***p < 0.001, ****p < 0.0001. Significance tested for each 5-min block by Student’s *t*-test between genotypes

Consistent with previously reported outcomes in adult rabbits and rats [18, 19, 35], exposures to severe hypoxia (8% O_2_) for 60 min resulted in significant increases in cFos expression within the NTS of control mice (Fig. 1A–B; *p* < 0.0001 for 8% O_2_ versus 21% O_2_) and TH-AMPK-α1/α2 knockouts (*p* < 0.05 for 8% O_2_ versus 21% O_2_). However, comparison of average counts (Fig. 1A–B) revealed no significant difference in the total number of cFos^+^ nuclei for TH-AMPK-α1/α2 knockouts when compared with controls (*p* = 0.336; 60 min, 8% O_2_). Nevertheless, in AMPK-α1/α2 knockouts, ventilatory responses to hypoxia (8% O_2_) were attenuated relative to controls for 60 min, matching the time period used to analyse cFos expression in NTS neurons. Minute ventilation of TH-AMPK-α1/α2 knockout mice (Fig. 1Ci) fell below normoxic values within 5 min, continued to rapidly decrease until 15 min (-34.1 ± 2.4%, *p* < 0.001 compared with controls) and thereafter remained significantly attenuated relative to controls for 60 min (*p* < 0.01 to *p* < 0.001). At 60 min, both breathing frequency (Fig. 1Cii) and tidal volume (Fig. 1Ciii) were significantly depressed in TH-AMPK-α1/α2 knockouts relative to AMPK-α1/α2 floxed controls (*p* < 0.01 for breathing frequency and *p* < 0.001 for tidal volume). This biphasic time course of the HVR is a feature of mice which exhibit an early acute response to hypoxia (first few minutes) that is characterised by hyperventilation due to increased ventilation. Thereafter, in steady-state hypoxia, mice express a temporal progressive decline in metabolism, a true hypometabolic response that is more pronounced than that observed in rats and different from the HVR in humans [70]. Therefore, ventilation decreases below normoxic values, but this still represents hyperventilation (i.e. an increase in the ventilatory equivalents (*V*E/*V*O_2_ and *V*E/*V*CO_2_), as shown in Figs. 7 and 8). Importantly in the context of this study, TH-AMPK-α1/α2 knockouts exhibit an exaggerated hypoventilation relative to controls.

We therefore compared by double-blind analysis, counts of cFos^+^ nuclei for each subnucleus of the NTS across all Bregma (-7.44 mm to -7.6 mm) that spanned the DAR identified by fMRI. Normalisation of cFos counts by surface area and section thickness ensured that differences in the size of subnuclei spanning multiple Bregma were accounted for. Moreover, to assess bilaterality, counts were analysed as the total number of cFos^+^ nuclei per 1000 μm^3^ for both sides of the NTS combined, and by right and left subdivisions. That said, exceptions were made for the AP and SolC, which in rodents and lagomorphs are midline structures [11, 45] and were, therefore, only analysed as a whole.

Using this strategy, we identified a small number of rostral NTS subnuclei that displayed significant and Bregma-specific reductions in hypoxia-induced cFos expression in TH-AMPK-α1/α2 knockouts relative to controls (Supplementary Fig. 1). Most notably, significant reductions in cFos expression of TH-AMPK-α1/α2 knockouts were identified within SolDL and SubP (Sub Postrema nucleus) at Bregma -7.48 mm, and in each case this was observed on the right side of the brainstem (SolDL, *p* < 0.05; SubP, *p* < 0.05) but not the left, in accordance with both the observed right-sided bilateral asymmetry and approximate anatomical location of the epicentre of the DAR identified by fMRI. Both SolDL and SubP have been proposed to innervate respiratory and cardiovascular compartments of the VLM. However, SolDL may primarily innervate compartments of the rostral VLM responsible for the modulation and/or generation of respiratory rhythmogenesis [3, 25, 39, 93], which could, at least in part, explain our finding that AMPK-α1/α2 deletion in catecholaminergic neurons selectively attenuates the HVR [56, 59].

As mentioned above, cFos expression in SolDL was significantly attenuated in TH-AMPK-α1/α2 knockouts relative to controls at Bregma -7.48 mm (*p* = 0.0154), but not in the next most caudal or rostral Bregma (Supplementary Fig. 2 Ai and Bi). By contrast, for SubP significant reductions in cFos expression were identified at Bregma -7.48 (*p* = 0.0294) and -7.56 mm (*p* = 0.0355; Supplementary Fig. 2 Aiv and Biv), the latter of which sits along this neuraxis at the caudal-most limit of the DAR identified by fMRI (Bregma -7.44 mm to -7.6 mm). Interestingly, combining the cFos^+^ counts of SubP across the three caudal-most Bregma of this neuraxis enhanced the degree of significance further (Bregma -7.48 mm to -7.56 mm: *p* = 0.0178). A similar pattern emerged for SolDL, whereby the highest degree of significance was achieved by combining the two rostral Bregma -7.44 mm and -7.48 mm (*p* = 0.0113).

**Fig. 2:**
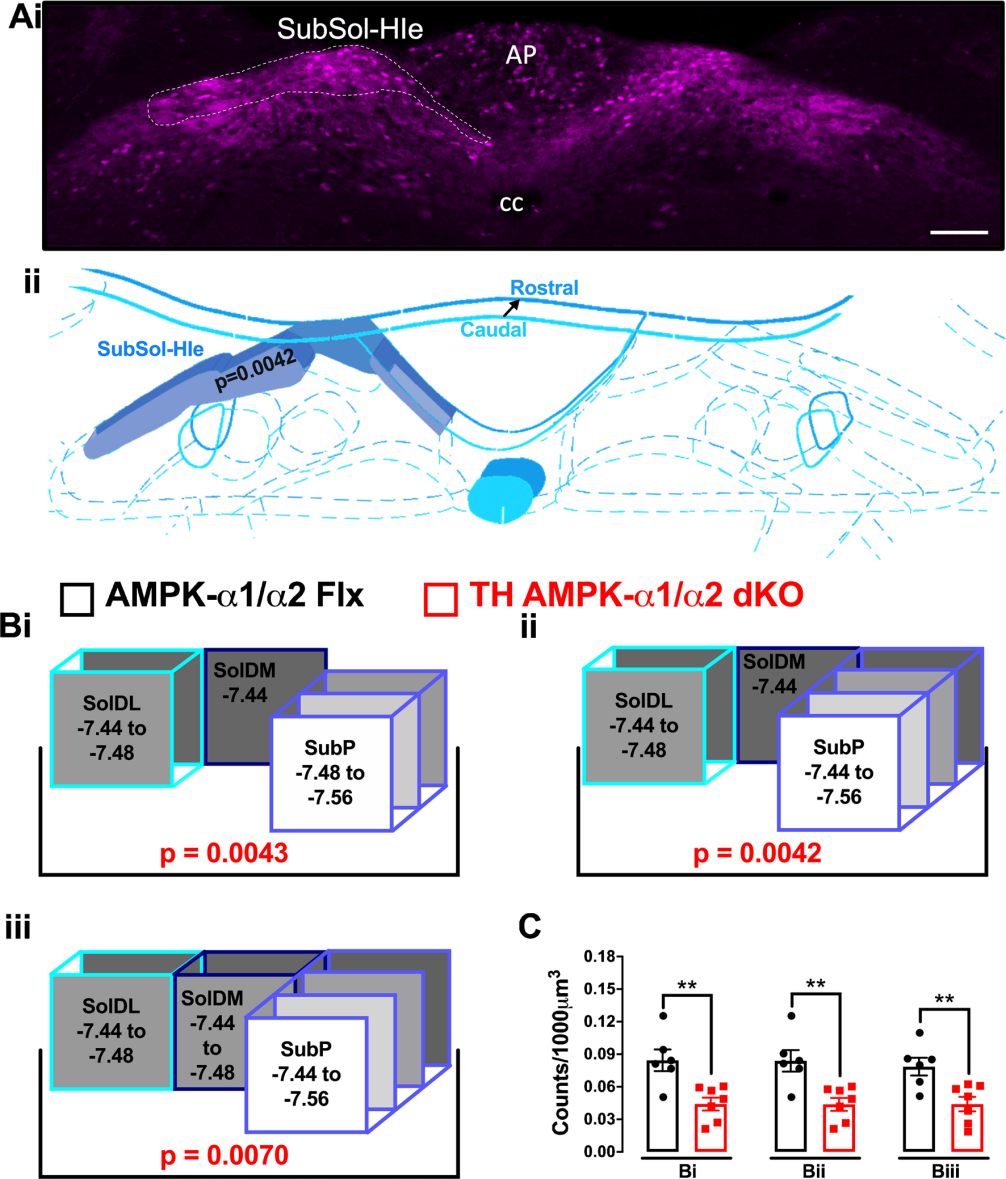
A map of the hypoxia-responsive subnucleus of the nucleus tractus solitarius as revealed by deficient cFOS expression following AMPK-α1/α2 deletion in catecholaminergic cells. **Ai** Exemplar confocal fluorescence image of brainstem section through the dorsal nucleus tractus solitarius (NTS) at the level of the area postrema (AP) and central canal (cc; approximate Bregma -7.44 mm) from a mouse line with TH-Cre driven expression of tdTomato (excitation 555 nm, emission 582 nm) from the Rosa26 locus. White dotted line indicates the approximate location of the AMPK-dependent hypoxia-responsive subnucleus of the NTS, named here as SubSol-HIe. Scale bar = 100 μm (see Supplementary Fig. 2 for whole brainstem section). **Aii** A 3D model of the predicted anatomical location of the hypoxia-responsive subnucleus of the NTS based on the highest degree of statistical significance obtained for comparison of cFOS counts in (**B**–**C**). **B** Schematic caudo-rostral (front to back) squares describe the process of mapping by statistical significance different combinations of SolDL, SolDM and SubP by Bregma that may shape the hypoxia-responsive subnucleus of the nucleus tractus solitarius (SubSol-HIe) with the *p* values shown in red for comparison of counts for cFos positive nuclei of AMPK-α1/α2 knockouts relative to controls. **C** Bar charts and scatter plots show mean ± SEM of cFos counts (per 1000 μm^3^) for AMPK-α1/α2 floxed (AMPK-α1/α2 Flx, black, *n* = 6 mice) versus TH-Cre driven AMPK-α1/α2 knockout mice (TH AMPK-α1/α2 dKO, red, *n* = 7 mice) for each combination of SolDL, SolDM and SubP by Bregma in (**B**). **p < 0.01; significance tested by Student’s *t*-test between genotypes for each grouping

**Fig. 3:**
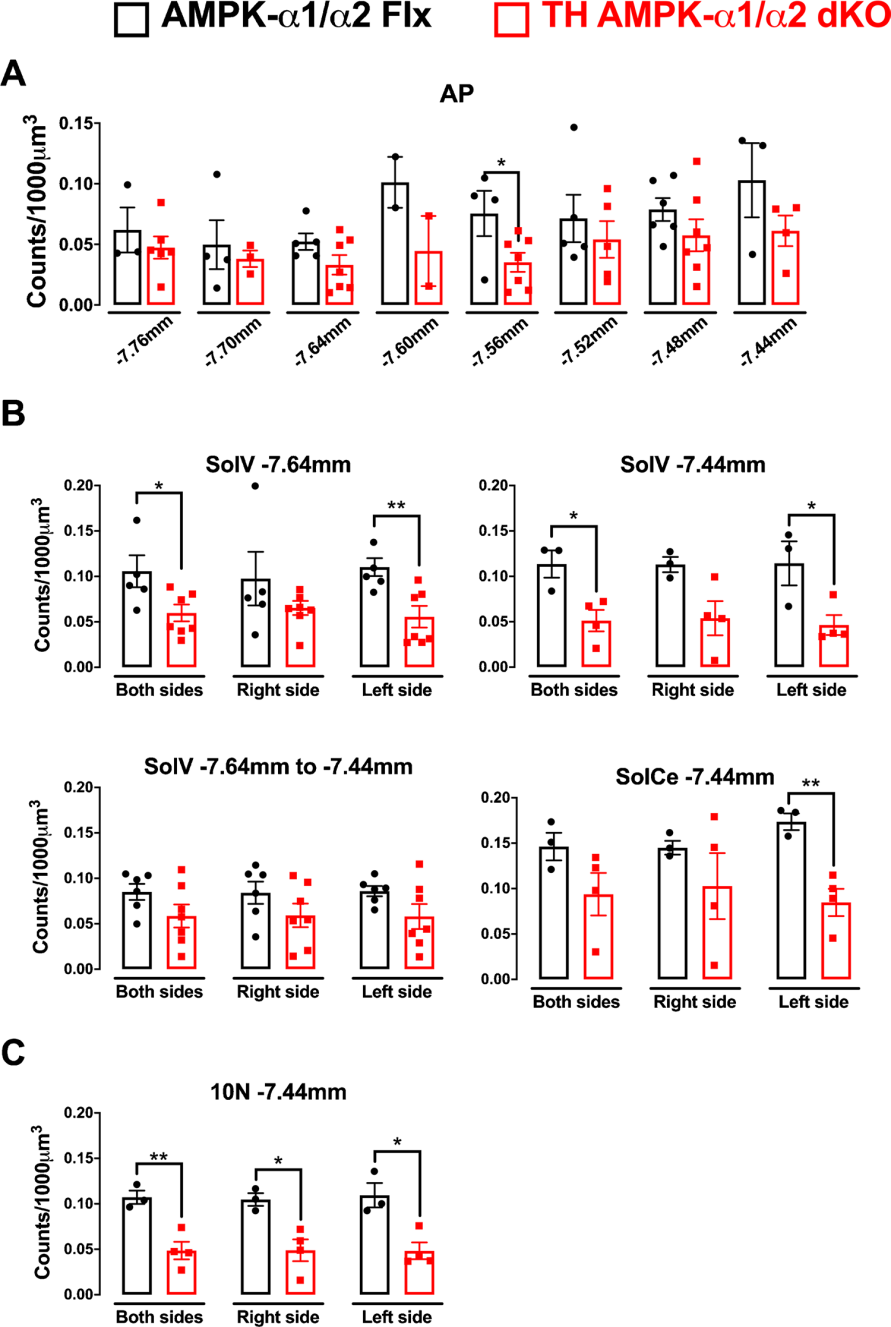
AMPK-α1/α2 deletion in catecholaminergic cells attenuates hypoxia-evoked cFos expression in additional nuclei of the nucleus tractus solitarius. Bar charts and scatter plots show means ± SEM for comparison of hypoxia-evoked cFos counts (per 1000 μm^3^) for additional nuclei of the nucleus tractus solitarius (NTS) in AMPK-α1/α2 floxed (AMPK-α1/α2 Flx, black) versus mice with AMPK-α1/α2 deletion in catecholaminergic cells (TH AMPK-α1/α2 dKO, red). Significant differences were found **A** for the area postrema (AP, a midline structure) at one Bregma (-7.56), **B** with left-sided bilateral asymmetry for SolV at two isolated Bregma (-7.64 and -7.44) and SolCe at one Bregma (-7.44) and **C** bilaterally for 10N at one Bregma (-7.44). AP = area postrema; SolV = ventral subnucleus; SolCe = central subnucleus; 10N = dorsal motornucleus of the vagus. *p < 0.05, **p < 0.01; significance tested by Student’s *t*-test between genotypes for each grouping

This presented a paradox, because SolDL and SubP are not connected anatomically. Therefore, we considered the possibility that SolDM (dorsomedial nucleus of the solitary tract) may be involved (Fig. 2), given that SolDM neurons may aid coordination of orofacial movements via the nucleus ambiguus [31], and all three nuclei incorporate TH expressing cells (Fig. 2A and Supplementary Fig. 3). We first examined cFos^+^ counts along the entire neuraxis of the murine SolDM, which spans a distance that ranges from Bregma -7.44 mm to -7.50 mm [28] but identified no statistical differences for any of these Bregma individually when comparing TH-AMPK-α1/α2 knockouts to controls (Supplementary Fig. 4A–B), nor when any of the adjacent Bregma were combined. Nevertheless, in order to thoroughly cross-compare all possible different combinations, we tested various models that included each SolDL, SolDM and SubP combination, starting with those that delivered the highest statistical difference for cFos^+^ counts in response to hypoxia (Fig. 2B–C). To our surprise, this resulted in the highest degree of statistical significance observed yet (*p* = 0.0043). Furthermore, the addition of SubP at Bregma -7.44 mm to this model marginally strengthened rather than weakening the degree of significance (*p* = 0.0042). Therefore, it would appear that the combination of SolDL, SolDM and SubP, all of which contribute to cardiorespiratory control during hypoxia (Supplementary Fig. 5), at Bregma -7.44 mm, and an extension for SolDL across two (-7.44 mm to -7.48 mm) and SubP across four (-7.44 mm to -7.56 mm) Bregma in a caudal direction most likely corresponds to the nucleus that exhibits reduced hypoxia-evoked neuronal activation in TH-AMPK-α1/α2 knockouts (Fig. 2C). Strikingly, both the anatomical location and right-side dominant bilateral asymmetry of this hypoxia-responsive nucleus (Supplementary Fig. 1), named here as SubSol-HIe (Sub(P) Sol(DL/M) hypoxia-induced expiration), within the caudal brainstem identified by cFos expression is consistent with the anatomical range, location and right-side dominant bilateral asymmetry of the DAR identified by fMRI (Bregma -7.1 mm to -7.6 mm). A deficit in cFos expression was also observed within the AP, but this was only apparent at Bregma -7.56 mm and did not extend further in either direction along the neuraxis of the DAR (Fig. 3A). That said a role for this segment of the AP should not be discounted lightly.

The aforementioned findings are all the more intriguing, because extended whole-brain analysis of our previously published fMRI data [59] identified further regions of interest (Supplementary Fig. 6) that lay both caudal and rostral to SubSol-HIe which exhibited significantly lower signal change (*p* < 0.005) during hypoxia in AMPK-α1/α2 knockouts than in AMPK-α1/α2 floxed controls. These regions of interest correspond by location, caudal to rostral, to the (1) nucleus retroambiguus [44], (2) ventrolateral medulla, (3) rostral ventral respiratory column (e.g. Bötzinger complex, retrotrapezoid nucleus/parafacial respiratory group) [6, 24, 37, 52] and (4) lateral nucleus of the cerebellum [54, 83, 86], and all are in receipt of noradrenergic inputs from the NTS and contribute to respiratory pattern formation (see “Discussion” section for further details).

Adding to the above, significant reductions in cFos^+^ neurons were seen in other nuclei of TH-AMPK-α1/α2 knockouts (Fig. 3) that, with the exception of the AP, were either left-side dominant (Fig. 3B) or bilateral (Fig. 3C), not previously registered by fMRI and located too lateral or too ventral to comprise any part of the hypoxia-responsive subnucleus identified by fMRI. Briefly, bilateral reductions in cFos^+^ neurons were identified for 10N at Bregma -7.44 mm (*p* < 0.01 for both sides, *p* < 0.05 for the right and left sides alone). Left-sided bilateral asymmetry was observed for SolCe at Bregma -7.44 mm (*p* < 0.01 for the left side alone) and SolV at Bregma -7.64 mm (*p* < 0.05 for both sides, *p* < 0.01 for left side alone) and Bregma -7.44 mm (*p* < 0.05 for both sides and left side alone); however, deficits in cFos counts within SolV were not enhanced by any combination of Bregma along this neuraxis.

### AMPK deletion in catecholaminergic cells inhibits hypoxia-evoked reductions in expiration time and increases in sigh frequency

Downstream of the NTS, hypoxia-evoked afferent input responses impact the parafacial nucleus which reduces expiration time (active expiration) to facilitate increases in breathing frequency [38] and the retrotrapezoid nucleus/parafacial respiratory group which coordinates sighs, that may conserve residual lung volume by reinflating collapsed alveoli [14, 52, 92]. Therefore, we next investigated the effect of AMPK-α1/α2 deletion on these ventilatory activities.

We found no significant difference with respect to the inspiration time ratio of hypoxia to normoxia (Ti) during exposures to 8% O_2_ for TH-AMPK-α1/α2 knockouts (0.9 ± 0.03 at 30 s; 1.4 ± 0.07 at 10 min; *n* = 37 exposures from 15 mice) relative to controls 0.9 ± 0.02 at 30 s; 1.3 ± 0.05 at 10 min; *n* = 54 exposures from 21 mice). That said, during hypoxia, the ratio of tidal volume (Tv)/Ti increased in TH-AMPK-α1/α2 knockouts to a peak at ~ 30 s (1.1 ± 0.03) that was comparable to controls (1.2 ± 0.04), Tv/Ti, then declined to a plateau (0.6 ± 0.03) that was significantly lower than controls (0.8 ± 0.03, *p* < 0.0001). Therefore, TH-AMPK-α1/α2 knockouts may not be able to maintain the same level of inspiratory effort during prolonged hypoxia.

**Fig. 4:**
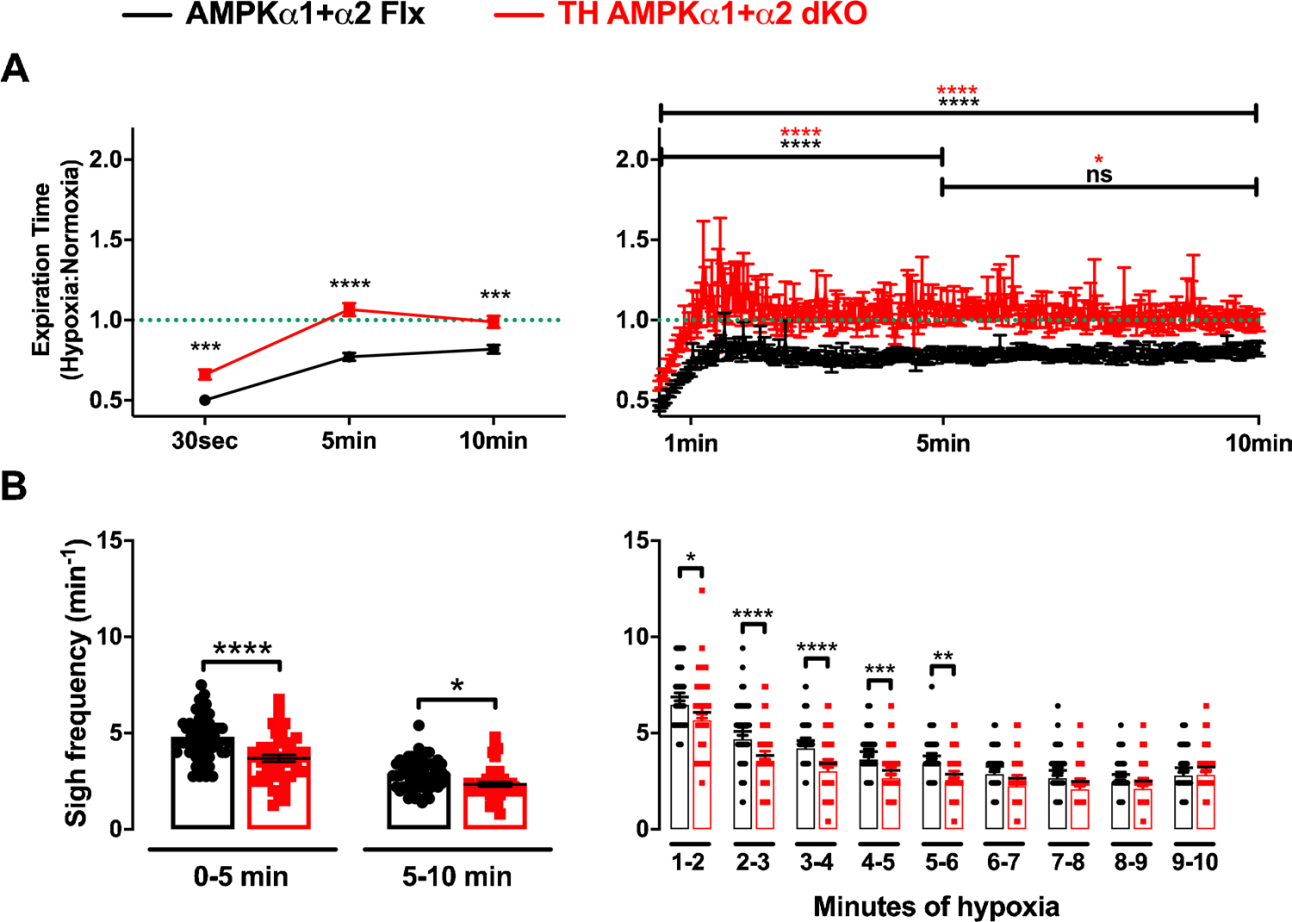
Deletion of AMPK-α1/α2 in catecholaminergic cells inhibits hypoxia-evoked reductions in expiration time and attenuates sigh frequency during the hypoxic ventilatory response.** A** Ratiometric changes relative to normoxia (green dotted line) of expiration time (mean ± SEM) during 10-min exposures to severe hypoxia (8% O_2_) at three selected time points (left) and at 2-s intervals (right) for AMPK-α1/α2 floxed mice (AMPK-α1/α2 Flx, black, *n* = 54 exposures from 21 mice) and for mice with AMPK-α1/α2 deletion in catecholaminergic neurons (TH-AMPK-α1/α2 dKO, red, *n* = 37 exposures from 15 mice). **B** Bar charts and scatter plots show mean ± SEM for sigh frequency during the first and second half (left) and every 60 s (right) during 10 min exposures to 8% O_2_ for AMPK-α1/α2 Flx (*n* = 58 exposures from 25 mice) and TH-AMPK-α1/α2 dKO mice (*n* = 46 exposures from 22 mice). ns = not significant, *p < 0.05, **p < 0.01, ***p < 0.001, ****p < 0.0001. Significance tested by two-way ANOVA with Sidak post hoc tests

In stark contrast, we observed marked attenuation of hypoxia-evoked reductions in expiration time in TH-AMPK-α1/α2 knockouts relative to controls (Fig. 4A). During exposures to 8% O_2_, the expiration time ratio of hypoxia to normoxia (Te) in AMPK-α1/α2 floxed mice displayed a marked reduction 30 s after the onset of hypoxia (0.5 ± 0.02). Thereafter, a lengthening of Te occurred until approximately 2 min followed by a secondary minor decrease and plateau, measuring 0.8 ± 0.02 by 5 min (*p* < 0.0001 compared with 30 s) and 0.8 ± 0.03 by 10 min (*p* < 0.0001 compared with 30 s, not significant compared with 5 min). The Te of TH-AMPK-α1/α2 knockouts was significantly lower relative to controls at 30 s (0.7 ± 0.03; *p* < 0.001) and lengthened over time, but unlike control mice, the prolongation of expiration time plateaued close to normoxic values, measuring 1.1 ± 0.04 by 5 min (*p* < 0.0001 compared with 30 s; *p* < 0.0001 compared with controls) and 1 ± 0.04 by 10 min (*p* < 0.05 compared with 5 min; *p* < 0.001 compared with controls). Consequent to attenuation of hypoxia-evoked reductions in Te, but not Ti, we observed increases in total breath time during hypoxia (0.7 ± 0.03 at 30 s; 1.1 ± 0.03 at 5 min;1.1 ± 0.04 at 10 min) relative to controls (0.6 ± 0.02 ms at 30 s (*p* < 0.05); 0.9 ± 0.02 at 5 min (*p* < 0.0001); 0.9 ± 0.02 at 10 min *p* < 0.0001). In short, mice with TH-AMPK-α1/α2 deletion in catecholaminergic neurons failed to maintain accelerated (likely active) expiration times and instead rapidly returned to and remained at Te and To values equivalent to those measured during normoxia that were significantly lengthened compared with controls at both 5 min and 10 min.

Sigh frequency was also significantly attenuated in TH-AMPK-α1/α2 knockouts compared with controls (Fig. 4B) during both halves of 10 min exposures to severe hypoxia (0–5 min: *p* < 0.0001; 5–10 min: *p* < 0.05). However, analyses at 1-min intervals revealed that although sigh frequencies were on average lower in TH-AMPK-α1/α2 knockouts during every minute except the last, the difference only reached significance during the first 5 min of exposures to 8% O_2_ compared with AMPK-α1/α2 floxed mice (*p* < 0.05 to 0.0001).

Similar results were obtained for expiration time and sigh frequency when grouped for males and females (Supplementary Fig. 7), although for females, reductions in hypoxia-evoked sigh frequency in TH-AMPK-α1/α2 knockouts did not reach significance between 5 and 10 min.

### AMPK deletion in catecholaminergic cells triggers oscillating apnoeic salvos during hypoxia

**Fig. 5:**
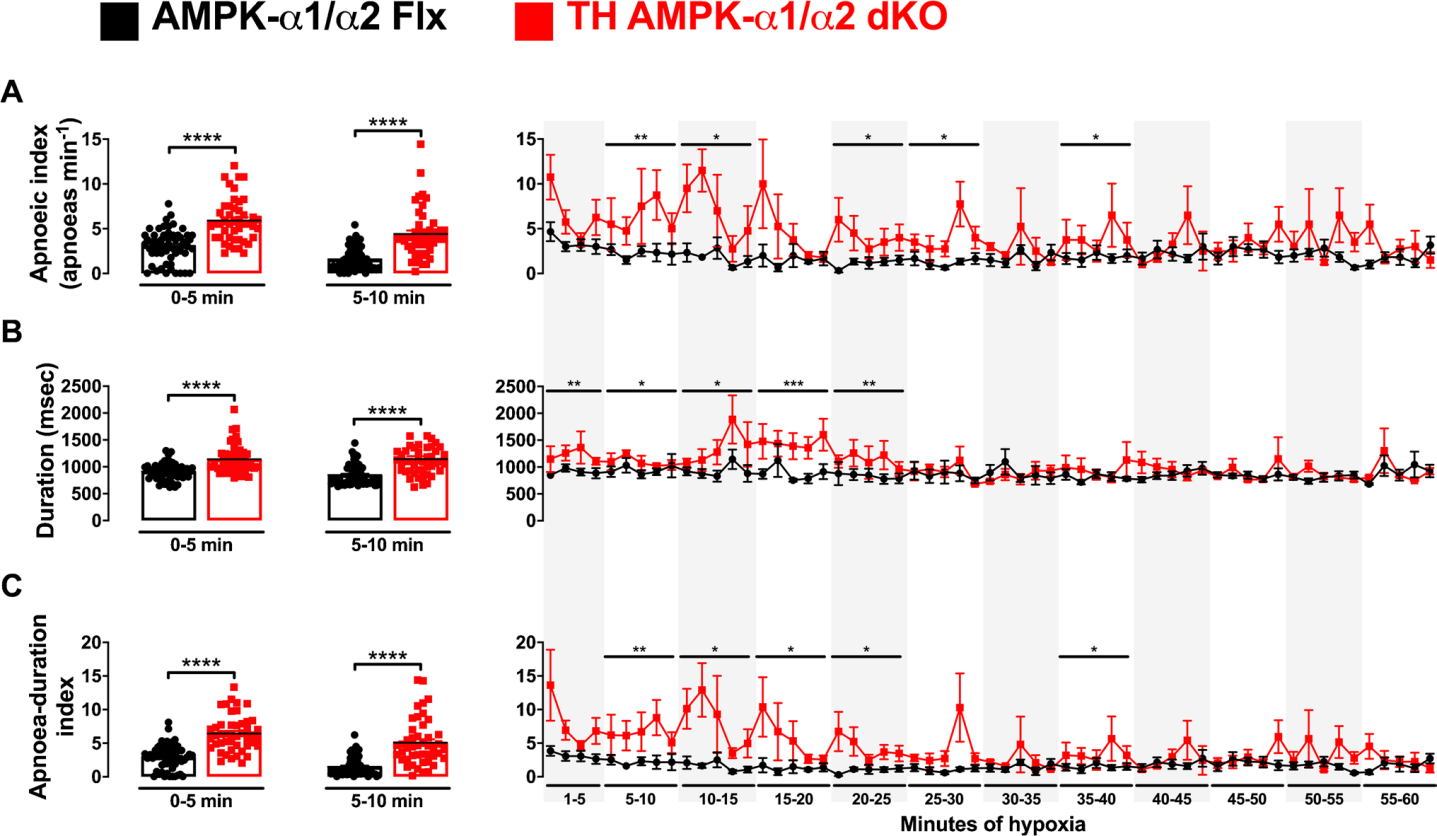
Hypoxia induces periodic salvos of apnoeas in mice with conditional deletion of AMPK-α1/α2 in catecholaminergic cells. Bar charts and scatter plots show mean ± SEM for the **A** apnoeic index (apnoeas min^−1^), **B** apnoea duration (msec) and **C** apnoea-duration index during the first and second 5-min blocks of 10-min exposures to hypoxia (left), and (right) whole minute averages during 60-min exposures to severe hypoxia (8% O_2_) in AMPK-α1/α2 floxed mice (AMPK-α1/α2 Flx, black, 10 min: *n* = 58 exposures from 25 mice, 60 min: *n* = 6 mice) and in mice with AMPK-α1/α2 deletion in catecholaminergic cells (TH-AMPK-α1/α2 dKO, red, 10 min: *n* = 46 exposures from 22 mice, 60 min: *n* = 4 mice). *p < 0.05, **p < 0.01, ***p < 0.001, ****p < 0.0001. Significance tested by Student’s *t*-tests (left panels) and two-way ANOVA with Sidak post hoc tests (right panels)

Consistent with previous observations [59], TH-AMPK-α1/α2 knockouts also exhibited a marked increase in apnoea frequency, apnoea duration and apnoea duration index compared with controls during 10-min exposures to hypoxia (Fig. 5A–C), and similar results were obtained when grouped for males and females (Supplementary Fig. 8). Moreover, minute-by-minute analyses during 60-min exposures to hypoxia revealed periodic “apnoeic salvos” in TH-AMPK-α1/α2 knockouts, which were characterised by time-dependent phasic increases in the number of apnoeas that occurred at a frequency of ~ 3–4 mHz and had a salvo duration of ~ 1 min (Fig. 5A). These apnoeic salvos were present in each of the four TH-AMPK-α1/α2 knockouts analysed (Supplementary Fig. 9), which were dampened by a time-dependent decline in apnoea duration in TH-AMPK-α1/α2 knockouts to a level equivalent to controls (Fig. 5B) that also dampened, as one might expect, oscillations in apnoea duration index of TH-AMPK-α1/α2 knockouts (Fig. 5C). By contrast, AMPK-α1/α2 floxed mice maintained a steady apnoea frequency throughout 60-min exposures to hypoxia.

### Attenuation of the HVR in mice by AMPK deletion in catecholaminergic cells is not related to differences in whole-body metabolic rate responses to hypoxia

**Fig. 6:**
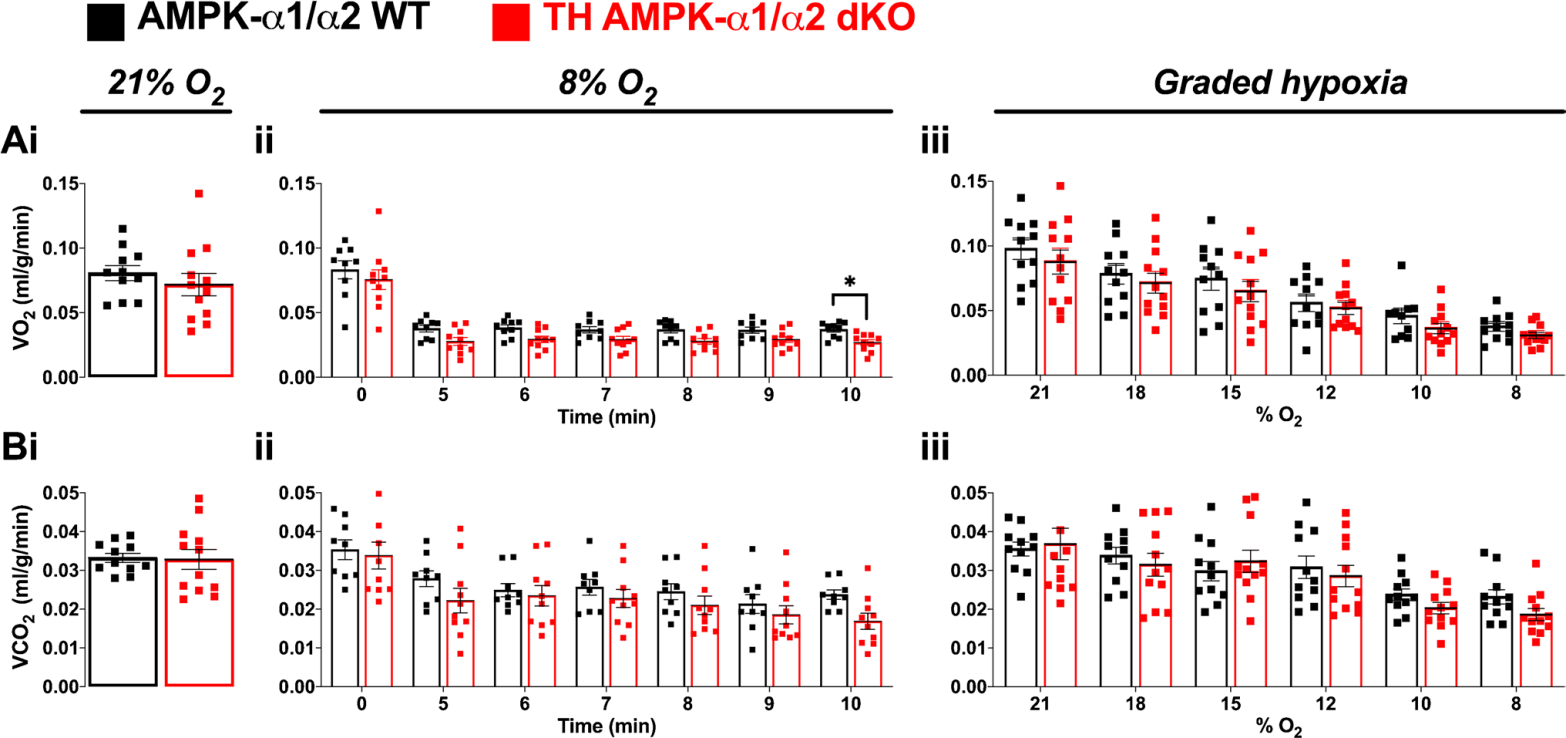
Metabolic responses during hypoxia are unaffected by AMPK-α1/α2 deletion in catecholaminergic cells. Bar charts and scatter plots show means ± SEM for **A** oxygen consumption (*VO*_*2*_) and **B** carbon dioxide production (*VCO*_*2*_) during (i) normoxia (21% O_2_), (ii) before (0) and during 5–10 min of 8% O_2_ and (iii) 5-min exposures to graded hypoxia (21% O_2_, 18% O_2_, 15% O_2_, 12% O_2_, 10% O_2_, 8% O_2_) in AMPK-α1/α2 wildtype mice (WT; black, 21% O_2_ *n* = 11 mice; 8% O_2_ *n* = 9 mice; graded hypoxia, *n* = 11 mice) and in mice with AMPK-α1/α2 deletion in catecholaminergic cells (TH-AMPK-α1/α2 dKO, red, 21% O_2_ *n* = 12 mice; 8% O_2_ *n* = 10 mice; graded hypoxia: *n* = 12 mice). Significance tested by Student’s *t*-tests between genotypes (Ai and Bi) and two-way ANOVA with Sidak post hoc tests (all other graphs)

The fact that TH-AMPK-α1/α2 knockouts exhibit a torpor-like state during hypoxia [59] concomitant with the observed ventilatory depression is indicative of mice entering a hypometabolic state [33] rather than hypoventilation per se. We therefore sought to assess the metabolic responses of control AMPK-α1/α2 wildtype and TH-AMPK-α1/α2 knockouts during hypoxia. Metabolic rates during normoxia (21% O_2_) were comparable between control AMPK-α1/α2 wildtype and TH-AMPK-α1/α2 knockouts for both oxygen consumption (*V*O_2_; Fig. 6Ai) and carbon dioxide production (*V*CO_2_; Fig. 6Bi).

To examine the acute metabolic response to hypoxia, which is associated with a relative hypoventilation in TH-AMPK-α1/α2 knockouts compared with controls, we first assessed a step change from 21 to 8% O_2_. During 10-min exposures to 8% O_2_ (Fig. 6Aii & Bii), control AMPK-α1/α2 wildtype mice displayed a reduction in *V*O_2_ relative to normoxia by 5 min, which was maintained until the end of the exposure at 10 min. Likewise, *V*CO_2_ was reduced relative to normoxia in control mice by 5 min, and this reduction was maintained until 10 min. Likewise, the *V*O_2_ of TH-AMPK-α1/α2 knockouts displayed similar responses relative to controls. Similarly, *V*CO_2_ was slightly reduced but not significantly different compared with control mice.

Furthermore, in order to compare the temporal metabolic response to hypoxia and determine if there was a difference in sensitivity to hypoxia between controls and TH-AMPK-α1/α2 knockouts, O_2_ consumption and CO_2_ production were assessed during 5-min exposures to graded hypoxia (5 min each of 21% O_2_, 18% O_2_, 15% O_2_, 12% O_2_, 10% O_2_, 8% O_2_; Fig. 6Aiii and Biii). A progressive reduction in O_2_ availability that lasted for a total of 25 min also revealed no differences between control and TH-AMPK-α1/α2 knockouts with respect to the gradual reductions of *V*O_2_ and *V*CO_2_, respectively.

**Fig. 7:**
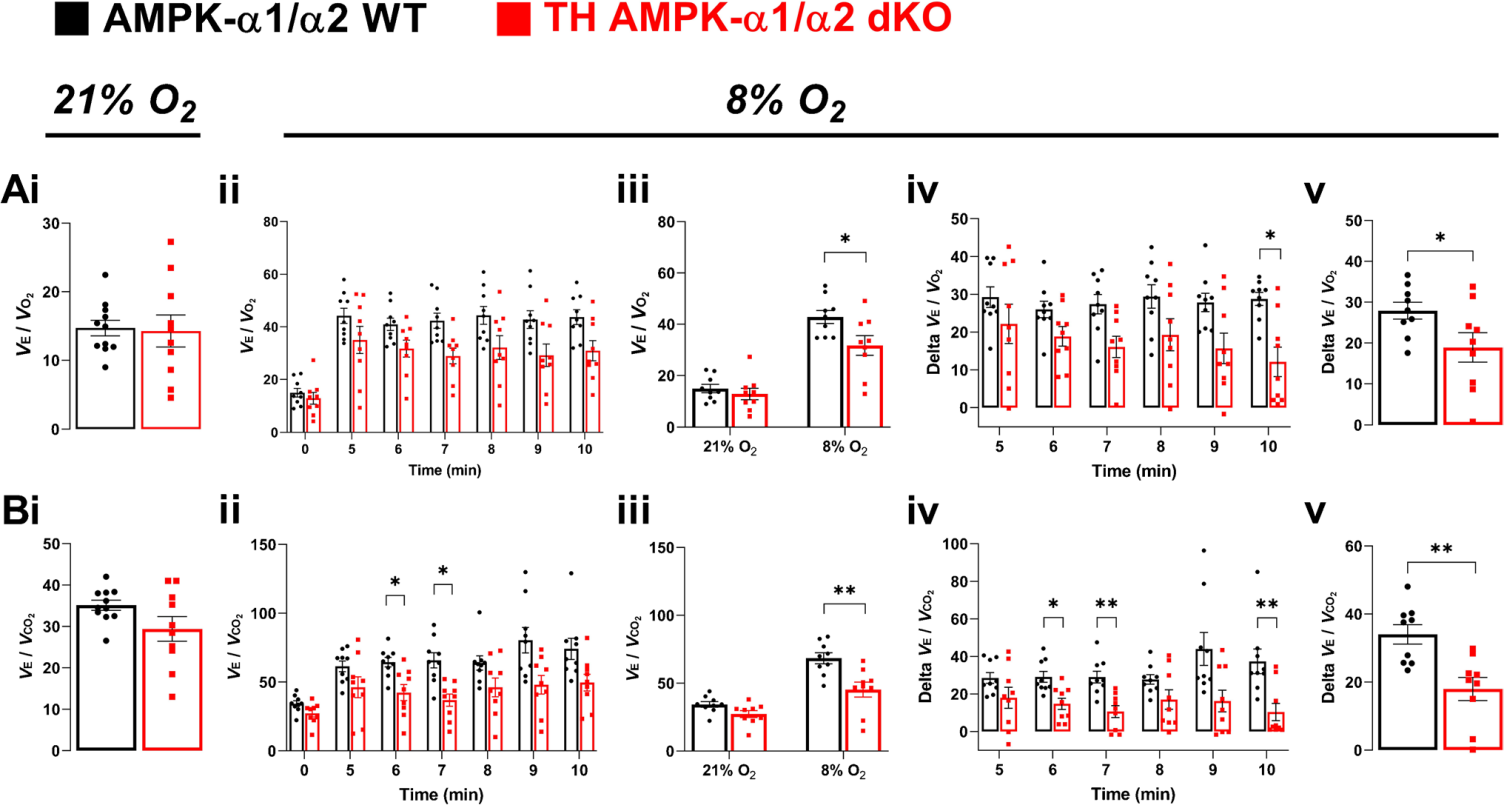
Ventilatory equivalents for oxygen and carbon dioxide during normoxia and acute exposure to hypoxia. Bar charts and scatter plots show Means ± SEM for **A** ventilatory equivalent for oxygen (*V*E/*V*O_2_) and **B** ventilatory equivalent for carbon dioxide (*V*E/*V*CO_2_) during (i) normoxia (21% O_2_), (ii) before (0) and during 5–10 min of 8% O_2_, (iii) before (21% O_2_) and during 8% O_2_ (average data for 5-10 min), (iv) change in ventilatory equivalent from baseline during minutes 5–10 of 8% O_2_ and (v) change in ventilatory equivalent from baseline for the average response to 8% O_2_ in AMPK-α1/α2 wildtype (WT; black, 21% O_2_ *n* = 11 mice; 8% O_2_ *n* = 9 mice) and in mice with AMPK-α1/α2 deletion in catecholaminergic cells (TH-AMPK-α1/α2 dKO, red, 21% O_2_ *n* = 10 mice; 8% O_2_ *n* = 9 mice. * = *p* < 0.05, ** = *p* < 0.01). Significance tested by Student’s *t*-tests (Ai, Bi, Av and Bv) and two-way ANOVA with Sidak post hoc tests (all other graphs)

**Fig. 8:**
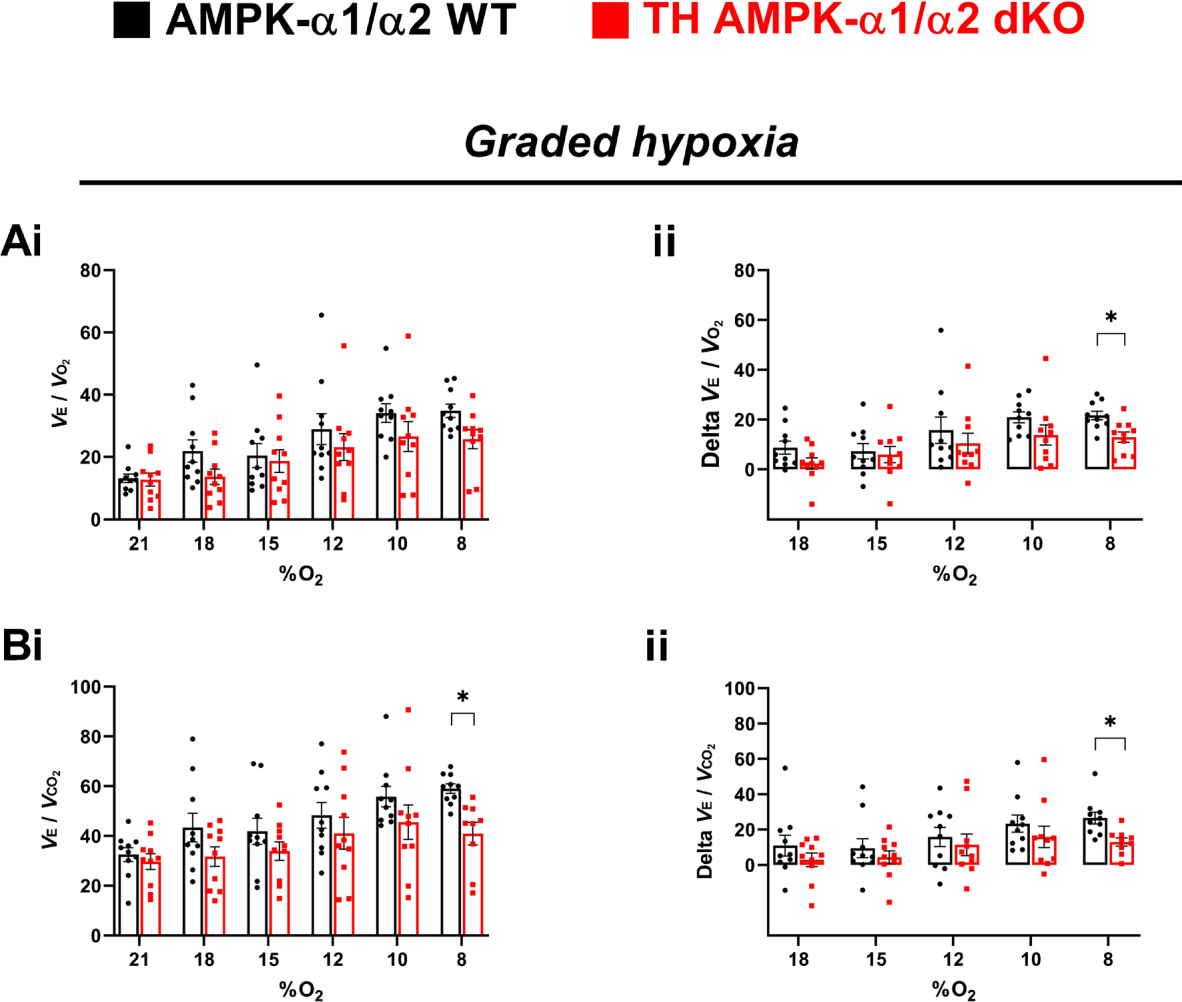
Ventilatory equivalents for oxygen and carbon dioxide during normoxia and exposure to graded hypoxia. Bar charts and scatter plots show means ± SEM for **A** ventilatory equivalent for oxygen (*V*E/*V*O_2_) and **B** ventilatory equivalent for carbon dioxide (*V*E/*V*CO_2_) during (i) normoxia (21% O_2_; 5 min) and the final minute of a 5-min exposure to 18%, 15%, 12%, 10% and 8% O_2_ and (ii) change in ventilatory equivalent from baseline in AMPK-α1/α2 wildtype mice (WT; black, *n* = 10) and in mice with AMPK-α1/α2 deletion in catecholaminergic cells (TH-AMPK-α1/α2 dKO, red, 21% O_2_: *n* = 10 mice. *p < 0.05. Significance tested by two-way ANOVA with Sidak post hoc tests

Concurrent assessment of ventilation and metabolism in control and TH-AMPK-α1/α2 knockout mice during step and graded challenges to hypoxia allowed for determination of the ventilatory equivalents, which importantly allows for consideration of the ventilatory response to hypoxia contextualised to metabolism, an especially important consideration in mice given their capacity to decrease metabolism in response to hypoxia. The ventilatory equivalents for oxygen (*V*E/*V*O_2_) and carbon dioxide (*V*E/*V*CO_2_) increased in response to hypoxic challenges (step and graded) in control and TH-AMPK-α1/α2 knockout mice demonstrating hyperventilatory responses to hypoxia (Figs. 7 and 8). However, hyperventilatory responses to hypoxia were considerably blunted in TH-AMPK-α1/α2 knockout mice, revealed as significantly decreased values for *V*E/*V*O_2_ and *V*E/*V*CO_2_ during step (Fig. 7Aii-iv and Bii-iv) and graded challenges (Fig. 8Ai and Bi). Moreover, assessments of the delta *V*E/*V*O_2_ and *V*E/*V*CO_2_, which reflect the true change in ventilation in response to hypoxia from baseline, confirmed that ventilatory responses to hypoxia are considerably reduced in TH-AMPK-α1/α2 knockout mice (Fig. 7Av and Bv and Fig. 8Aii and Bii). Responses were similar in male and female mice (Supplementary Figs. 10–13), and therefore data were pooled.

### Deletion of AMPK in catecholaminergic neurons does not alter brainstem catecholamine content

**Fig. 9:**
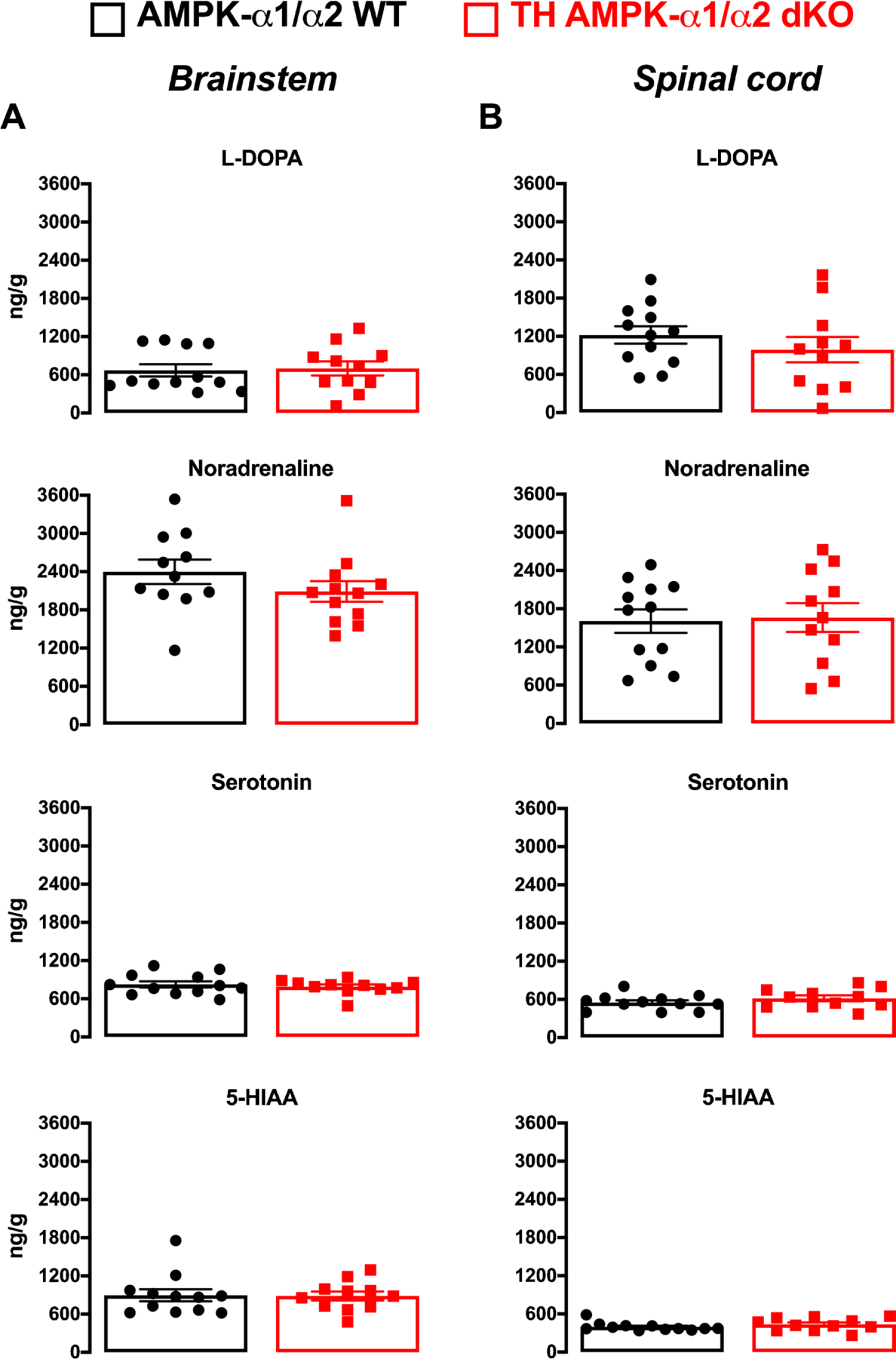
Deletion of AMPK-α1/α2 catalytic subunits in catecholaminergic cells did not alter bioamine content of the brainstem or spinal cord. Bar charts and scatter plots show mean ± SEM of bioamine levels within the **A** brainstem and **B** spinal cord of AMPK-α1/α2 wildtype (WT; black, *n* = 11 mice) and in mice with AMPK-α1/α2 deletion in catecholaminergic cells (TH AMPK-α1/α2 dKO, red, *n* = 12 mice). 5-HIAA = 5-hydroxyindoleacetic acid. Significance tested by Student’s *t*-test between genotypes for each bioamine

Finally, we sought to assess the bioavailability of catecholamines within the central nervous system at the sites targeted by conditional AMPK deletion in catecholaminergic neurons, in males only to insure against any confounding variable due to the oestrus cycle. Bioamine levels within the brainstems and spinal cords (Fig. 9) of control AMPK-α1/α2 wildtype and TH-AMPK-α1/α2 knockouts revealed no differences in catecholamine/monoamine levels, namely noradrenaline, serotonin, the catecholamine precursor L-DOPA or the serotonin metabolite 5-hydroxyindoleacetic acid (5-HIAA).

## Discussion

Using cFos expression as a surrogate marker for neuronal activation, we have mapped a novel AMPK-dependent hypoxia-responsive subnucleus of the NTS, namely SubSol-HIe, which is in receipt of chemoafferent inputs from the carotid body [25]. AMPK deletion in catecholaminergic neurons selectively attenuated, with right-sided bilateral asymmetry, activation during hypoxia of this neuronal cluster, which partially spans three anatomical nuclei of the nucleus tractus solitarius (NTS), namely SolDL (Bregman -7.44 mm to -7.56 mm), SolDM (Brema -7.44 mm) and SubP (Bregma -7.4 mm to -7.56 mm) and perhaps also the area postrema (AP; at Bregma -7.56 mm). Strikingly, this approximates both the anatomical location and, with the exception of the AP which is a midline structure, the right-side dominant bilateral asymmetry observed for the AMPK-dependent hypoxia-responsive subnucleus of the NTS previously identified by fMRI [59]. The functional significance of observed right-left asymmetry of brainstem activation by hypoxia is unclear. However, such asymmetry is evolutionarily conserved and may provide specialisation sufficient to prevent delays in responses to stressful stimuli [9, 88], such as hypoxia, by limiting conflicting outputs from each side of the brain, as has been previously described in relation to cognitive performance [15, 87]. Accordingly, like most other NTS subnuclei, both SolDL and SubP have been proposed to innervate respiratory and cardiovascular compartments of the VLM. However, SolDL may primarily innervate compartments of the rostral VLM responsible for the modulation and/or generation of respiratory rhythmogenesis [3, 25, 39, 93], which could, at least in part, explain our finding that AMPK-α1/α2 deletion in catecholaminergic neurons selectively attenuates the HVR [56, 59].

These findings are all the more intriguing, because extended whole-brain fMRI analysis identified regions of interest during hypoxia that lay both rostral and caudal to the dorsal active region (DAR) described previously [59], all of which exhibited significantly lower oxygen consumption (*p* < 0.005) during hypoxia in AMPK-α1/α2 knockouts, namely, from caudal to rostral, the nucleus retroambiguus which provides ascending inputs critical to inspiratory rhythm generation [44], the ventrolateral medulla which incorporate C1 adrenergic neurons that drive, for example, active expiration [3, 60, 71, 80], the rostral ventral respiratory column (e.g. Bötzinger complex, retrotrapezoid nucleus/parafacial respiratory group) which coordinates post-inspiratory activity and active expiration [6, 24, 37, 52] and the lateral nucleus of the cerebellum [86] which receives noradrenergic inputs from the NTS and contributes to eupnoeic breathing and orofacial rhythms [54, 83]; accordingly, AMPK deletion in adrenergic neurons through Cre expression via the phenylethanolamine N-methyl-transferase promoter (PNMT-Cre) does not attenuate the HVR [57]. Therefore, it is also notable that in addition to afferent inputs from the NTS to the nucleus retroambiguus [31, 44] contributing to the coordination of orofacial rhythms, neurons of both SolDM and the nucleus retroambiguus receive afferent inputs from the superior laryngeal nerve that contribute to both orofacial rhythms and the swallowing reflex [31, 77].

We also identified significant attenuation of cFos expression in subsections of three other NTS nuclei, namely SolV, SolCe and 10N, the anatomical location of which was too lateral and/or ventral to comprise any part of the hypoxia-responsive subnucleus identified by fMRI, and were perhaps too small to be revealed by this technique.

This aforementioned findings may be significant given that while AMPK-α1/α2 deletion attenuated increases in sigh frequency, because a few carotid body chemoafferent terminals project to 10N [25], hypoxia has been found to increase sigh frequency in a manner dependent on vagal in addition to carotid body chemoafferent activity [7]. The physiological significance of this being that sighs have been proposed to conserve residual lung volume by reinflating collapsed alveoli [14, 52, 92].

Furthermore, AMPK-α1/α2 deletion in catecholaminergic neurons attenuated hypoxia-evoked decreases in expiration time (and thus total breath time) which facilitate increases in breathing frequency. Breathing frequency during hypoxia is determined in great part by changes in the inter-breath interval [42] that primarily results from a shift from passive (elastic recoil of the lungs, and relaxation of respiratory muscles) to active expiration (myogenic acceleration of expiration) and consequent reductions in total breath time [1, 8, 42, 80]. This suggests that AMPK-α1/α2 deletion in catecholaminergic neurons attenuates hypoxia-evoked active expiration, although electromyographic recordings from abdominal and intercostal muscles will be required to confirm this. Nevertheless, it is notable that downstream of the carotid bodies, area postrema and the NTS, adrenergic C1 neurons of the ventrolateral medulla [3, 60, 71, 80] drive active expiration by activating the pFRG/RTN [1, 8, 60, 80], which in turn rhythmically modulates expiration via recruitment of abdominal and intercostal muscles [1, 65]. This is significant, because we previously demonstrated that AMPK-α1/α2 deletion in adrenergic cells (PNMT-Cre) does not block the HVR [57]. Therefore, AMPK-dependent afferent inputs from non-adrenergic, catecholaminergic (likely nor-adrenergic) neurons of SubSol-HIe may function in part to integrate and relay carotid body afferent input responses to the pFRG/RTN via C1 adrenergic neurons of the ventrolateral medulla, to reduce expiration time and thus total breath time in order to facilitate increases in ventilatory frequency.

Importantly, we further demonstrated that AMPK-α1/α2 deletion in catecholaminergic neurons had no impact on metabolic rate and state relative to controls during acute 10-min exposures to hypoxia (8% O_2_) or graded increases in hypoxia severity over a 30-min period (5 min each of 21% O_2_, 18% O_2_, 15% O_2_, 12% O_2_, 10% O_2_, 8% O_2_). This confirms that TH-AMPK-α1/α2 knockouts enter frank blunted ventilatory responses during hypoxia relative to controls, despite the fact that TH-AMPK-α1/α2 knockouts also enter a torpor-like state during hypoxia [59], which is sometimes associated with mice entering a hypometabolic state [33] concomitant with the observed ventilatory depression. Assessment of ventilation normalised to metabolism, that is, the ventilatory equivalents for oxygen (*V*E/*V*O_2_) and carbon dioxide (*V*E/*V*CO_2_) revealed that TH-AMPK-α1/α2 knockouts express a blunted hyperventilatory response to hypoxia, most clearly demonstrated in our analysis by evidence of significantly decreased changes in *V*E/*V*O_2_ and *V*E/*V*CO_2_ from baseline in response to hypoxia in TH-AMPK-α1/α2 knockouts compared with controls.

Furthermore, no differences in brainstem or spinal catecholamines or serotonin levels were observed in TH-AMPK-α1/α2 knockouts relative to controls, including therein noradrenaline, serotonin, the catecholamine precursor L-DOPA and the serotonin metabolite 5-hydroxyindoleacetic acid (5-HIAA). Therefore, deletion of AMPK-α1/α2 catalytic subunits in catecholaminergic neurons did not alter monoamine dynamics or result in pronounced hyper- or hypoplasia of TH-positive cells. Thus, it is unlikely that the breathing irregularities, relative hypoventilation and apnoeas observed during hypoxia were consequent to aberrant catecholaminergic neurotransmission per se. Emergence of relative hypoventilation and apnoea during severe poikilocapnic hypoxia [59] therefore suggests that AMPK gain of function may represent a major maturational element underlying the central nervous system component of the shift from neonatal HVR to adult HVR patterning [32]. Moreover, the ventilatory dysfunction observed in TH-AMPK-α1/α2 knockouts likely results from the failure of signal integration and relay of command outputs from the hypoxia-responsive NTS subnucleus identified here, namely SubSol-HIe, to the respiratory central pattern generators. This is most likely conferred by the loss of direct phosphorylation and regulation by AMPK of non-canonical targets such as ion channels [5, 13, 40, 46, 63, 67, 75], enzymes for transmitter biosynthesis [95], receptors [2] and transporters [78], the failure of AMPK-dependent developmental expression of such targets and/or failure of maturational changes in related synaptic control mechanisms. Accordingly, relative to wild-type C57BL/6 J mice, hypoxia sensitivity of the brainstem and apnoea frequency has been shown to be exacerbated in a mouse model of Rett syndrome (methyl-CpG binding protein 2 gene (Mecp2)-/y knockout) [47, 82], which is known to impact catecholaminergic neurons of the NTS.

Strikingly, the integrated HVR of the TH-AMPK-α1/α2 knockouts is highly reminiscent of HVRs observed in neonates [32], i.e. marked late hypoxic ventilatory depression culminating in apnoeic episodes, along with preservation of hypercapnic hypoxic ventilatory responses [59]. When considered alongside the emergence of apnoea during severe poikilocapnic hypoxia [59], this provides further support for the view that AMPK gain of function may represent a major maturational element underlying the central nervous system component of the shift from neonatal HVR to adult HVR patterning [32]. Conversely, AMPK deficiency might precipitate apnoea of prematurity, which contributes to cardiopulmonary arrest and respiratory failure in neonates [4, 64] (requiring longer-term intensive care) and SIDS [12], when apnoea of prematurity occurs in concert with cyclical tachypnoea and brief apnoea [12, 64]. In accordance with this and the site of SubSol-HIe within the NTS, it has been highlighted that with respect to SIDS, there is a critical gap in our knowledge regarding the integration and regulation of peripheral afferents and descending autonomic ventilatory control mechanisms at the NTS [4], where reduced chemoreceptor sensitivity and reduced CNS oxygenation [72] may contribute to dysregulation of respiratory rhythm and autoresuscitation failure [4, 17, 64]. This is all the more intriguing, because cell-specific AMPK deficiency accompanies obesity and type 2 diabetes [30, 76, 91], which represent maternal stressors strongly associated with SIDS [43, 84], and these stressors are also strongly associated with central sleep apnoea in adults [26, 62, 74, 79, 89]. Moreover, under anaesthesia, hypoxia triggers irreversible respiratory failure in TH-AMPK-α1/α2 knockouts [59], a known risk factor for adult humans suffering from sleep apnoea [10, 16].

Further studies on the AMPK-dependent hypoxia-responsive subnucleus of the NTS identified here, namely “SubSol-HIe”, are therefore required so that we can address the formidable question [32]: “What role and by what mechanism does AMPK play in the adaptive and maladaptive respiratory responses elicited in the neonate and adult by the wide range of potential hypoxic stimulus presentations, i.e., intermittent, sustained, chronic, etc.?”

### Supplementary information

Below is the link to the electronic supplementary material.

Supplementary file1 (PDF 9982 KB)

## Data Availability

All available data are included in this manuscript.
