# Supplementary material for: SubSol-HIe is an AMPK-dependent hypoxia-responsive subnucleus of the nucleus tractus solitarius that coordinates the hypoxic ventilatory response and protects against apnoea in mice
Source: Pflugers Arch. 2024 Apr 18;476(7):1087–107. doi: 10.1007/s00424-024-02957-6 (PMC11166843; doi:10.1007/s00424-024-02957-6)
Supplement: Supplementary file 1 — Supplementary file1 (PDF 9982 KB) [file 424_2024_2957_MOESM1_ESM.pdf]

Supplementary Materials

SubSol-HIe is an AMPK-dependent hypoxia-responsive subnucleus of the nucleus tractus solitarius that coordinates the hypoxic ventilatory response and protects against apnoea in mice

Sandy MacMillan[1], David P. Burns[2], Ken D. O'Halloran[2], A. Mark Evans[1*]

[1]Centre for Discovery Brain Sciences, Hugh Robson Building, University of Edinburgh, Edinburgh, EH8 9XD, UK. [2]Department of Physiology, School of Medicine, College of Medicine & Health, University College Cork, Cork, Ireland.

[*]**CORRESPONDING AUTHOR**: A. Mark Evans, Centre for Discovery Brain Sciences, College of Medicine and Veterinary Medicine, Hugh Robson Building, University of Edinburgh, Edinburgh, EH8 9XD, UK. E-mail: mark.evans@ed.ac.uk

Supplementary Methods

Functional Magnetic Resonance Imaging Mice were anaesthetised with 0.8-1.3% isoflurane in air, their body temperature was maintained at 37°C, and their breathing frequency was monitored using pressure pad sensors. A 7-Tesla horizontal bore magnetic resonance imaging scanner (Agilent Technologies, Yarnton, UK), equipped with a high-performance gradient insert (12-cm inner diameter, maximum gradient strength 400 mT/m) was used. A birdcage coil (72-mm diameter) delivered radiofrequency transmission with signal reception via a mouse two-channel phased array brain coil. All sequences were acquired with a field of view 19.2 x 19.2 mm with 30 contiguous coronal slices of 0.4-mm thickness. Structural images were acquired by fast spin echo sequence (train length, 8; repetition time, 3,100 ms; effective echo time, 36 ms; 8 signal averages; acquisition matrix, 19.2 x 19.2; zero filled to 256 x 256). Functional images (230 volumes) were acquired during normoxia (21% O_2) and hypoxia (8% O_2) using three-shot echo planar imaging sequences (repetition time, 6,000ms [2,000ms/shot]; effective echo time, 7.08ms; flip angle, 90 degrees; 1 signal average; acquisition matrix, 64 x 64). Bias-corrected (www.slicer.org) structural images were co-registered to a template (www.spmmouse.org) and averaged using SPM8 imaging software (Wellcome Trust Centre for Neuroimaging, University College London, London, UK). Functional data were realigned to mean volumes of each series, spatially normalized (SPM8 co-registration procedures) using each animal's structural

scan, and smoothed using a 0.7 x 0.7 x 4mm full width at half maximum Gaussian filter. For first level analysis and further information see[4].

In second-level (group) analysis, the t-map for contrast (knockout > control) was thresholded at a level of $p<0.005$ with a four-voxel cluster threshold (SPM8 imaging software). Between group differences were analysed using a region-of-interest tool. For regions with significant group differences, all voxel signals were averaged at each time point

Supplementary Figures

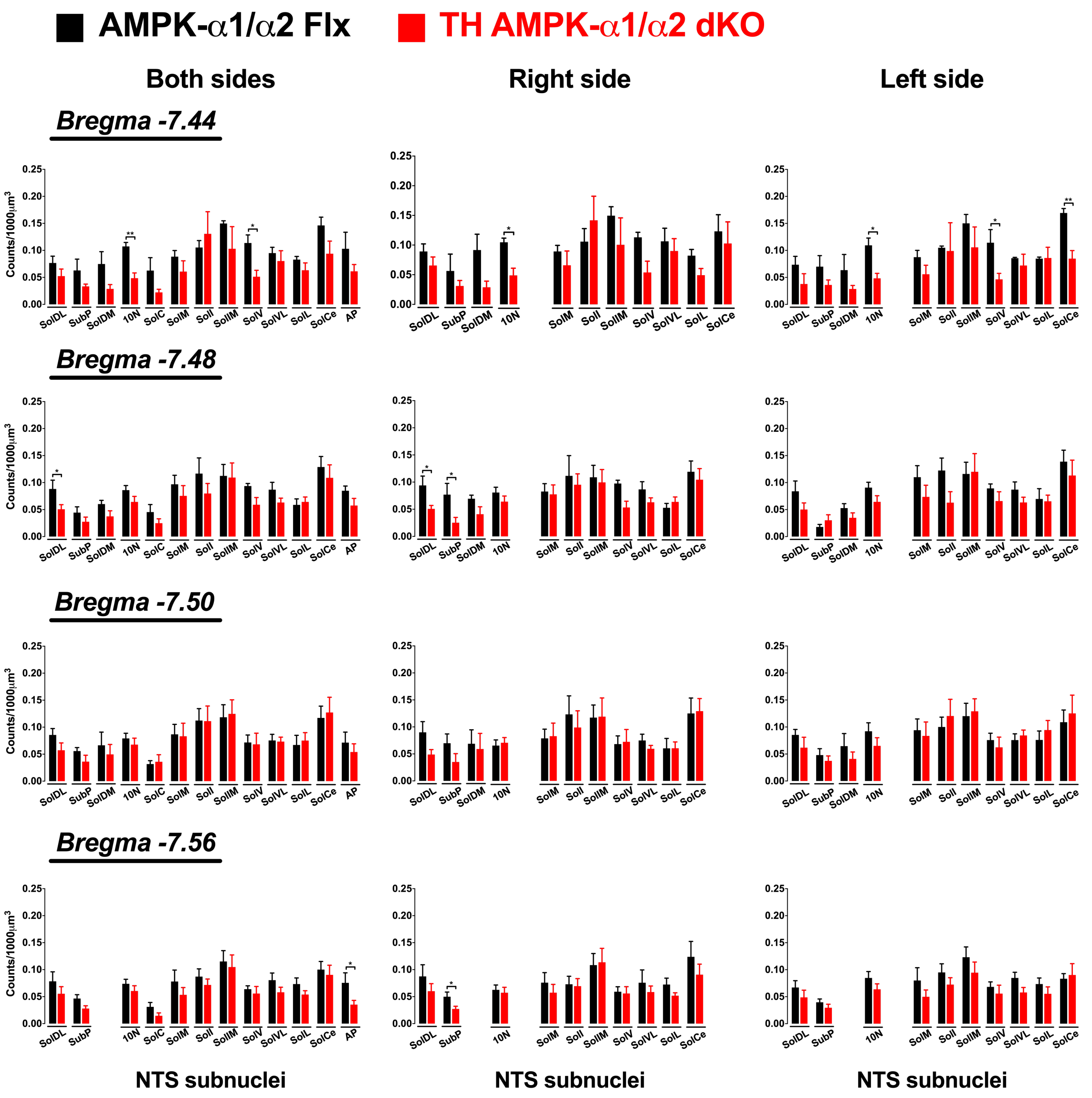

Supplementary Fig. 1: Separation of Bregma and subnuclei revealed significant attenuations of cFos expression following AMPK-α1/α2 catalytic subunit deletion in catecholaminergic cells. Means ± SEM of cFos counts (per $1000\mu m^3$) for the total (left panels), right (middle panels), and left (right panels) sides of all subnuclei within the nucleus tractus solitarius (NTS) in four brainstem Bregma in AMPK-α1/α2 floxed (AMPK-α1/α2 Flx, black) and TH-Cre driven AMPK-α1/α2 double knockout mice (TH AMPK-α1/α2 dKO, red). * = $p<0.05$, ** = $p<0.01$; significance tested by Student's t-test between genotypes for each grouping. In this and all subsequent figures AP = area postrema, cc = central canal, SubP, subpostrema nucleus, SolC = commissural division, SolM = medial nucleus, SolDL = dorsolateral nucleus, SolIM = intermediate nucleus, SolCe, central nucleus, SolI = interstitial nucleus, SolV = ventral nucleus, SolVL = ventrolateral nucleus, SolL = lateral nucleus.

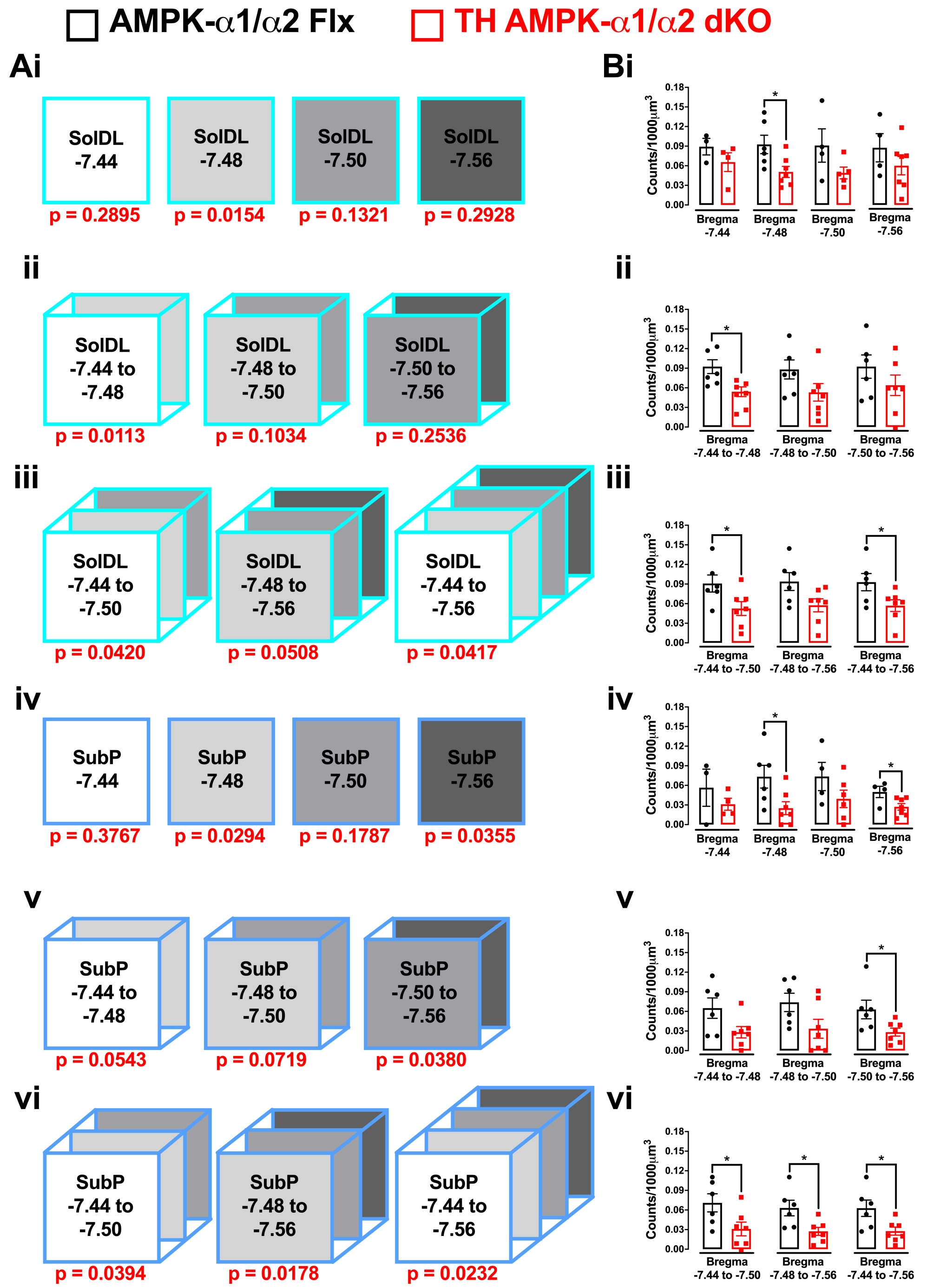

Supplementary Fig. 2: The deficiency of cFos expression in mice with targeted deletion of AMPK-α1 and -α2 catalytic subunits likely spans multiple Bregma at each identified subnucleus. (**A**) Rostro-Caudal (front to back) schematics representing which combination of SolDL and SubP in relation to which Bregma have been tested, and the p-values (red) that were obtained. (**B**) Means ± SEM of cFos counts (per 1000µm^3) for each Bregma combination of SolDL and SubP in AMPK-α1/α2 floxed (AMPK-α1/α2 Flx, black, n = 3-6 mice) and TH-Cre driven AMPK-α1 and -α2 double knockout mice (TH AMPK-α1/α2 dKO, red, n = 4-7 mice). * = p<0.05; significance tested by Student's t-test between genotypes for each grouping.

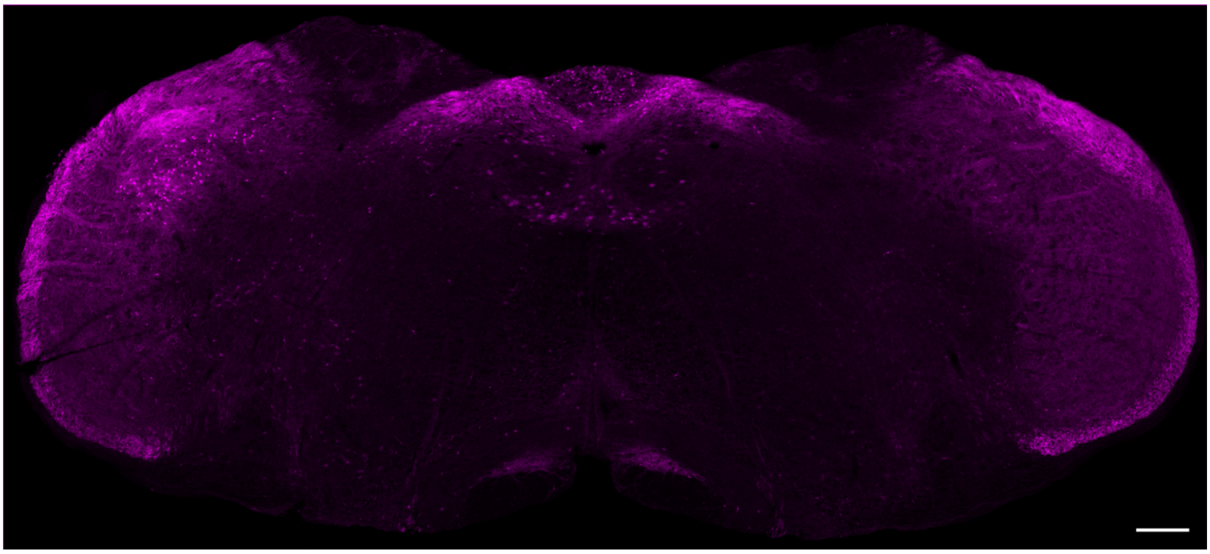

Supplementary Fig. 3: Exemplar whole brainstem confocal fluorescence image for a section through the dorsal nucleus tractus solitarius (NTS) at the level of the area postrema, central canal and the dorsal motor nucleus of the vagus (not indicated; approximate Bregma -7.48mm) from a mouse line with TH-Cre driven expression of tdTomato (excitation 555 nm, emission 582 nm) from the Rosa26 locus. Scale bar 100µm.

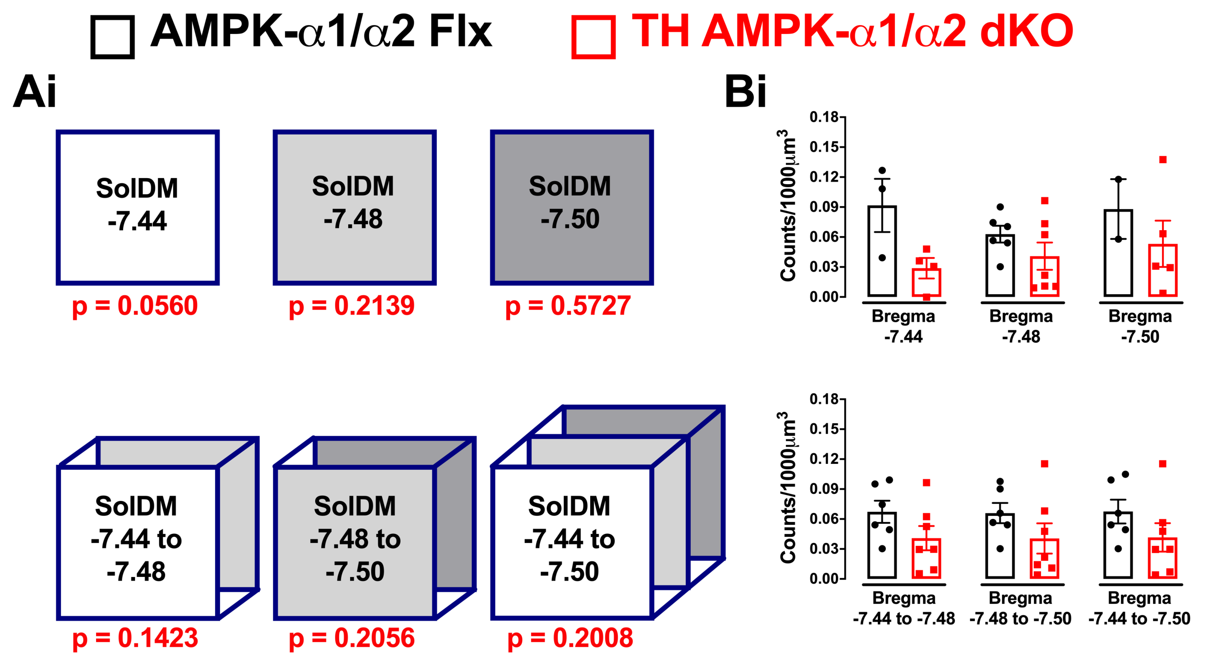

Supplementary Fig. 4: Assessment of the degrees of significance for attenuated cFos expression between genotypes within SolDM as a 'linker' nucleus at multiple Bregma. (**A**) Schematics representing which combination of SolDM in relation to the Bregma has been tested, and the p-values that were obtained. (**B**) Means ± SEM of cFos counts (per $1000\mu m^3$) for each combination from (A) for AMPK-α1/α2 floxed (AMPK-α1/α2 Flx, black, n = 2-6 mice) and TH-Cre driven AMPK-α1 and -α2 double knockout mice (TH AMPK-α1/α2 dKO, red, n = 4-7 mice). Significance tested by Student's t-test between genotypes for each grouping.

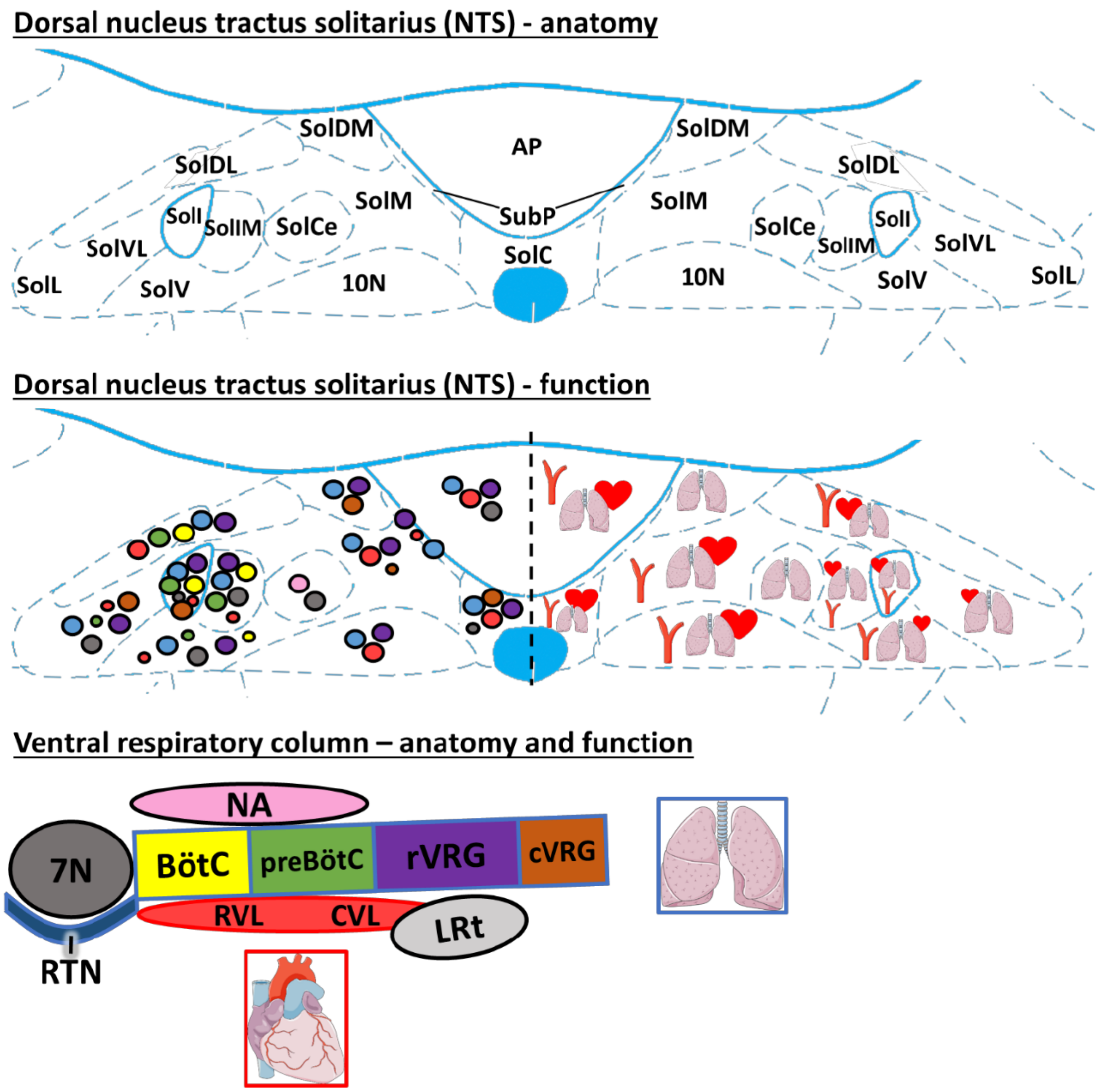

Supplementary Figure 5: **Schematic identifies cardiorespiratory projections of nuclei of the nucleus tractus solitarius.** Top panel shows the anatomical organisation of the nucleus tractus solitarius (NTS) according to the Mouse Brain Atlas at Bregma -7.48mm. Middle panel shows the functional organisation of the NTS according to findings of with respect to the physiological responses coordinated by their afferent inputs. Lower panel shows the compartmentalisation of the ventral respiratory column (VRC), which is colour coded according to the physiological function of each compartment (border colour – respiratory: blue, cardiovascular: red) and (middle panel) the afferent inputs from each NTS nucleus (colour matched). Larger circles within the NTS subnuclei indicate strong innervation of each colour coded compartment of the VRC, smaller circles indicate weaker innervation.

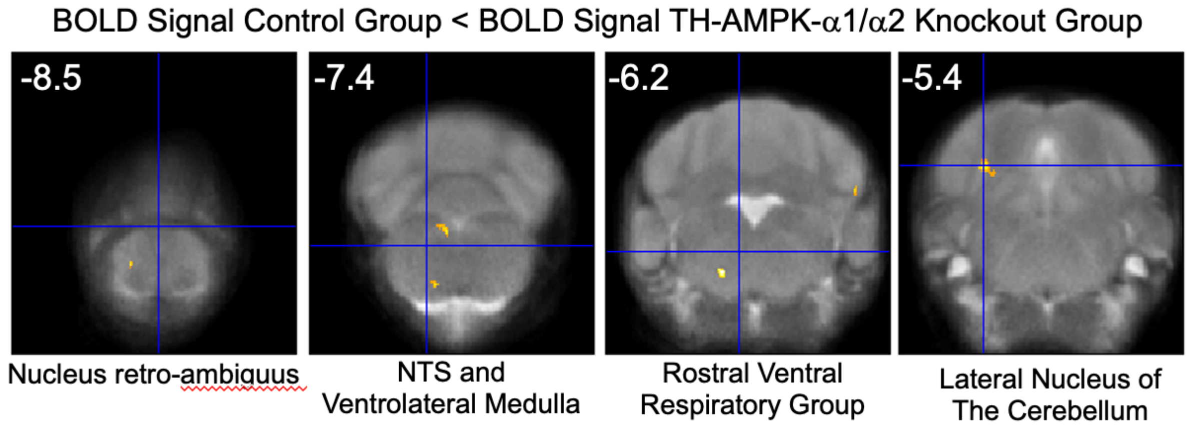

Supplementary Fig. 6: Functional magnetic resonance imaging identifies the wider AMPK-dependent, hypoxia-responsive respiratory circuit. Whole-brain fMRI identified regions rostral and caudal to the Dorsal Active Region (DAR) of the NTS (Bregma -7.4) that exhibited significantly lower signal change ($P<0.005$) during hypoxia in AMPK-α1/α2 knockouts than in AMPK-α1/α2 floxed control mice. The locations of these regions of interest highlighted in gold, correspond to, from caudal (left) to rostral (right): the nucleus retro-ambiguus, the ventrolateral medulla, the rostral ventral respiratory group and the lateral nucleus of the cerebellum.

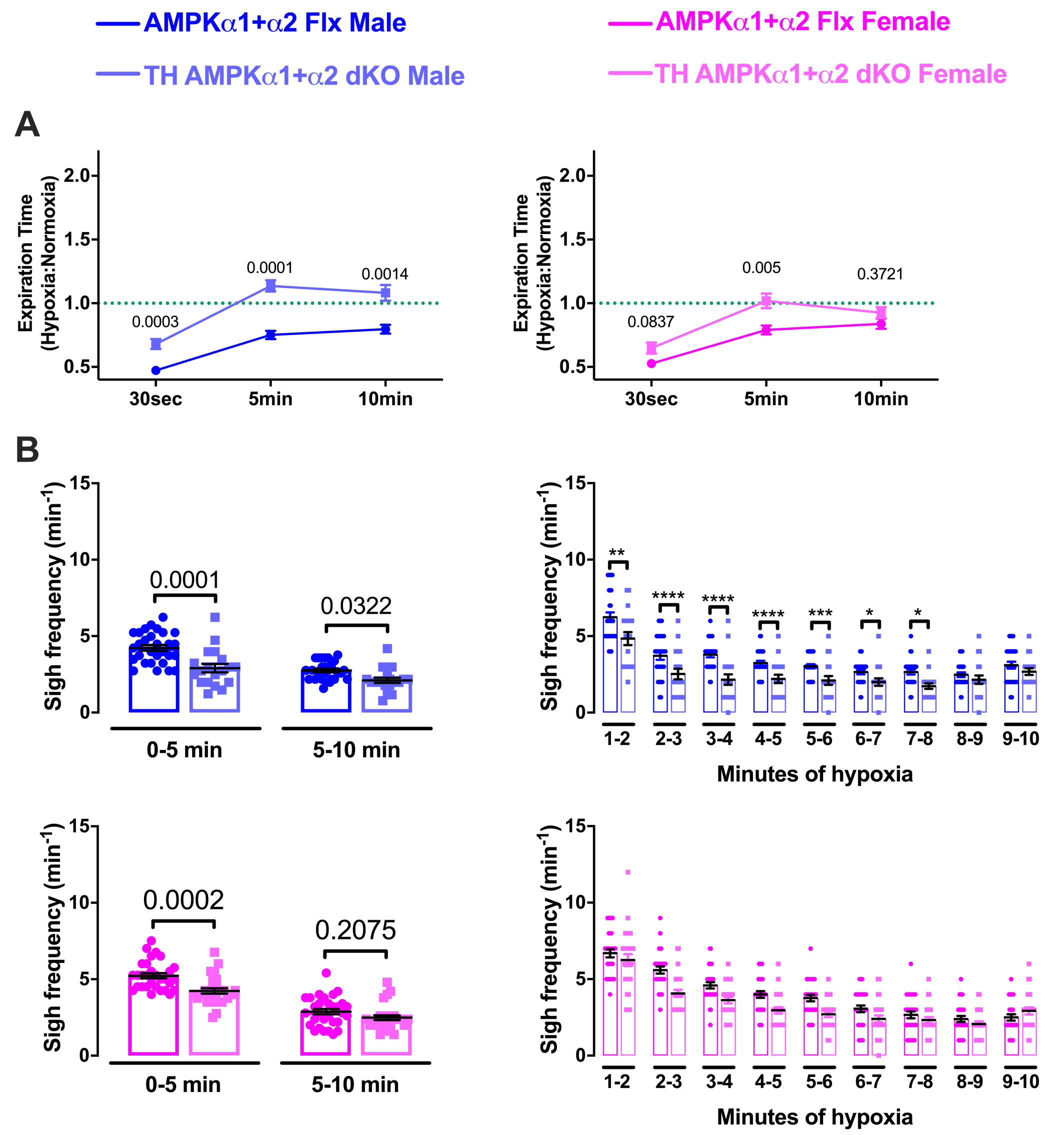

Supplementary Fig. 7: AMPK deletion in catecholaminergic neurons of males and females blocks hypoxia-evoked decreases in expiration time and attenuates increases in sigh frequency. The following data are shown for males (AMPK-α1/α2 floxed (Flx), dark blue; AMPK-α1/α2 knockout, light blue) and females (AMPK-α1/α2 floxed dark pink; AMPK-α1/α2 knockout light pink: (**A**) Ratiometric changes relative to normoxia (green dotted line) of expiration time (Mean ± SEM) during 10min exposures to severe hypoxia (8% O_2) at three selected time points for AMPK-α1/α2 Flx mice (n = 12 males, n = 9 females) and for mice with AMPK-α1/α2 deletion in catecholaminergic neurons (TH-AMPK-α1/α2 dKO: n = 7 males, n = 8 females). (**B**) Bar charts and scatter plots show Mean ± SEM for sigh frequency during the first and second half (left), and every 60 seconds (right) during 10min exposures to 8% O_2 for AMPK-α1/α2 Flx (n = 13 males, n = 12 females) and TH-AMPK-α1/α2 dKO mice (n = 10m, n = 12 females). ns = not significant. P-values shown above means or as * = p<0.05, ** = p<0.01, *** = p<0.001, **** = p<0.0001. Significance tested by two-way ANOVA with Sidak post-hoc tests.

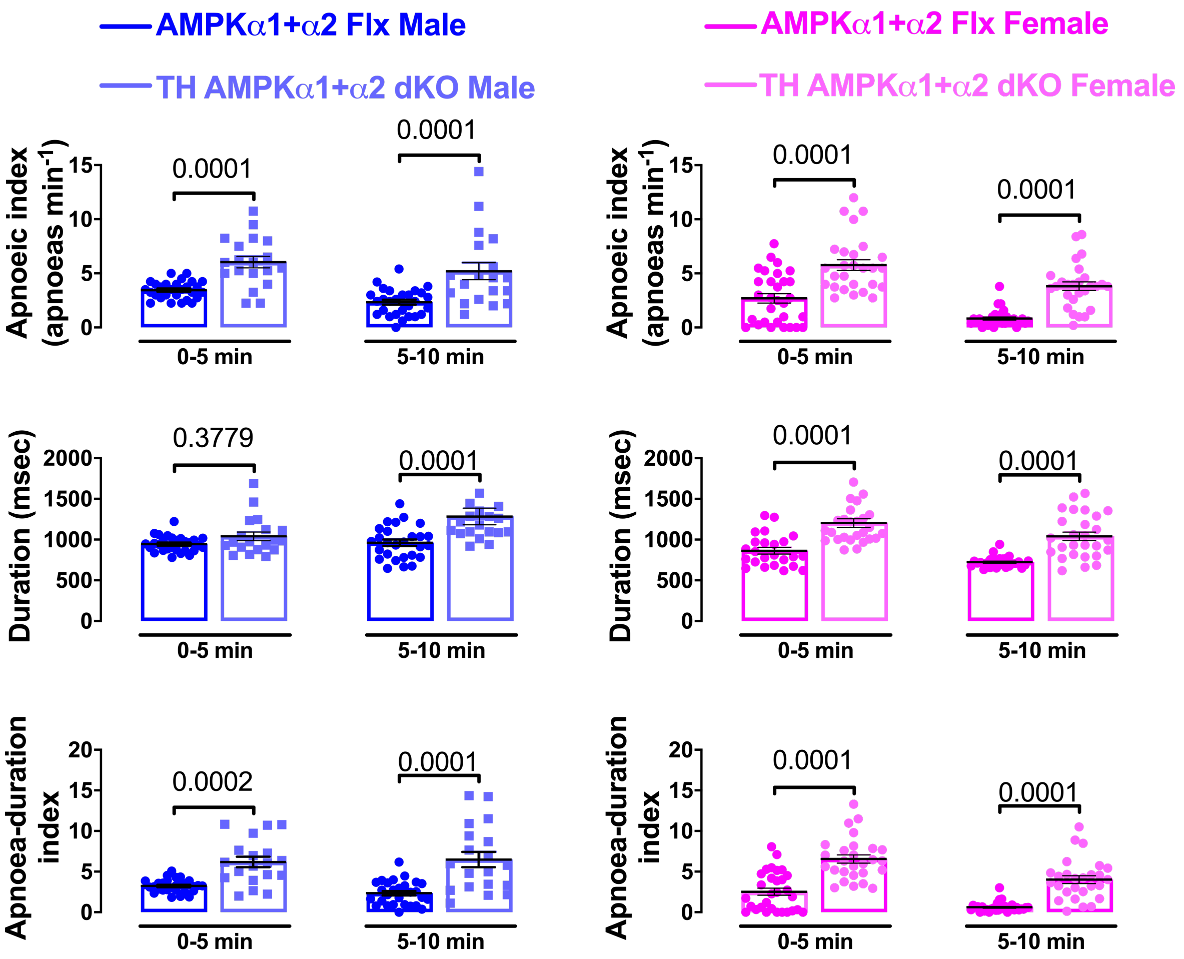

Supplementary Fig. 8: AMPK deletion in catecholaminergic neurons of males and females increase apnoeas during hypoxia. The following data are shown for males (AMPK-α1/α2 floxed (Flx), dark blue; AMPK-α1/α2 knockout, light blue) and females (AMPK-α1/α2 Floxed dark pink, AMPK-α1/α2 knockout, light pink): Bar charts and scatter plots of Mean ± SEM for the (**A**) apnoeic index (apnoeas min^{-1}), (**B**) apnoea duration (msec), and (**C**) apnoea-duration index during the first and second 5 minute blocks (left) of 10 minute exposures to severe hypoxia (8% O_2) in AMPK-α1/α2 Flx mice (n = 13 males, n = 12 females) and in mice with AMPK-α1/α2 deletion in catecholaminergic cells (TH-AMPK-α1/α2 dKO: n = 10m, n = 12 females). P values shown above bars. Significance tested by two-way ANOVA with Sidak post-hoc tests.

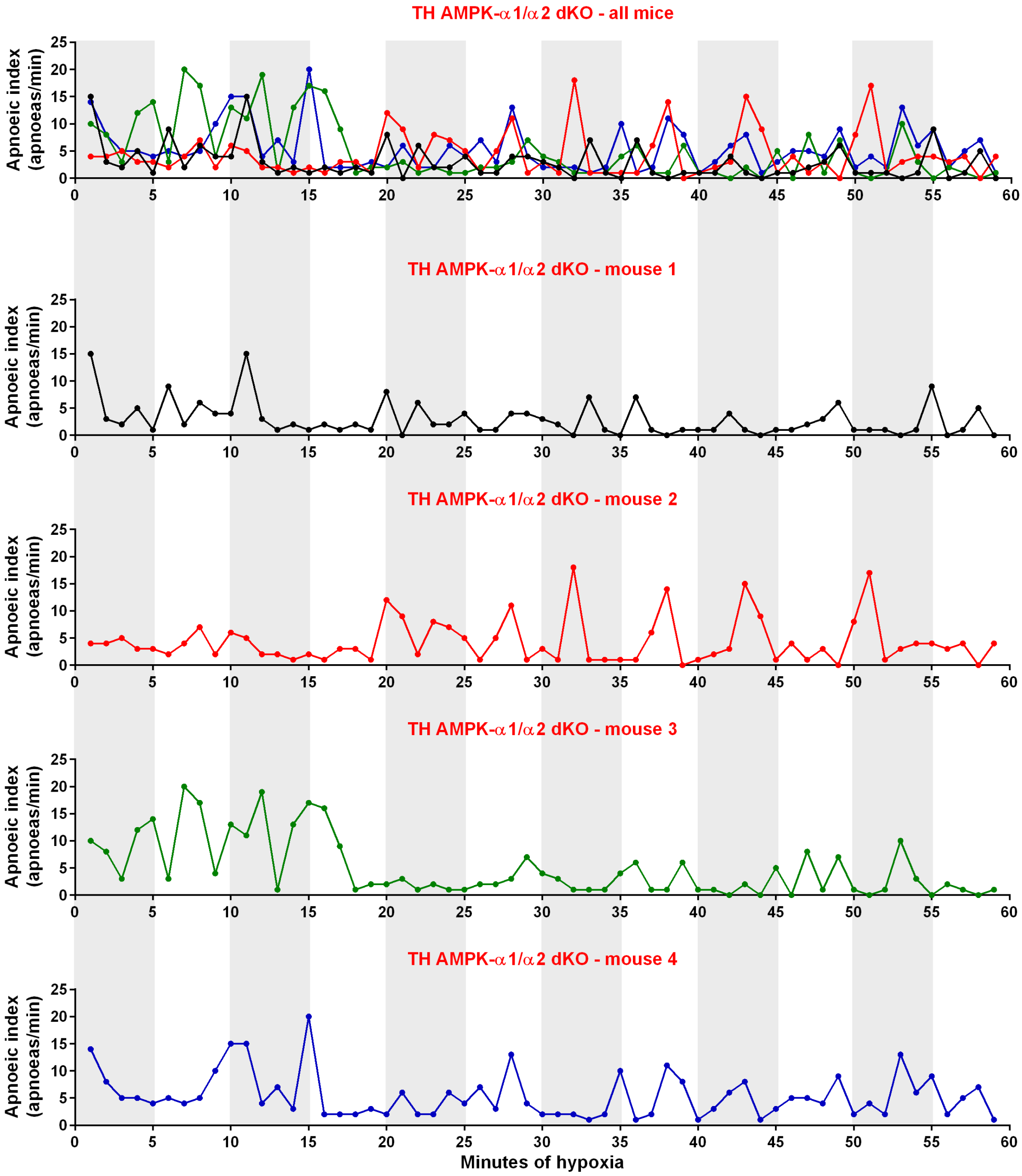

Supplementary Fig. 9: Dual deletion of the AMPK-α1/α2 catalytic subunits in catecholaminergic cells precipitates apnoeic salvos during prolonged exposures to severe hypoxia. Line charts show the mean apnoea index over a 60min period of severe hypoxia (8% O_2) for all experimental TH-Cre driven AMPK-α1/α2 double knockout mice (TH-AMPK-α1/α2 dKO, n = 4) at the top and each individual trace below.

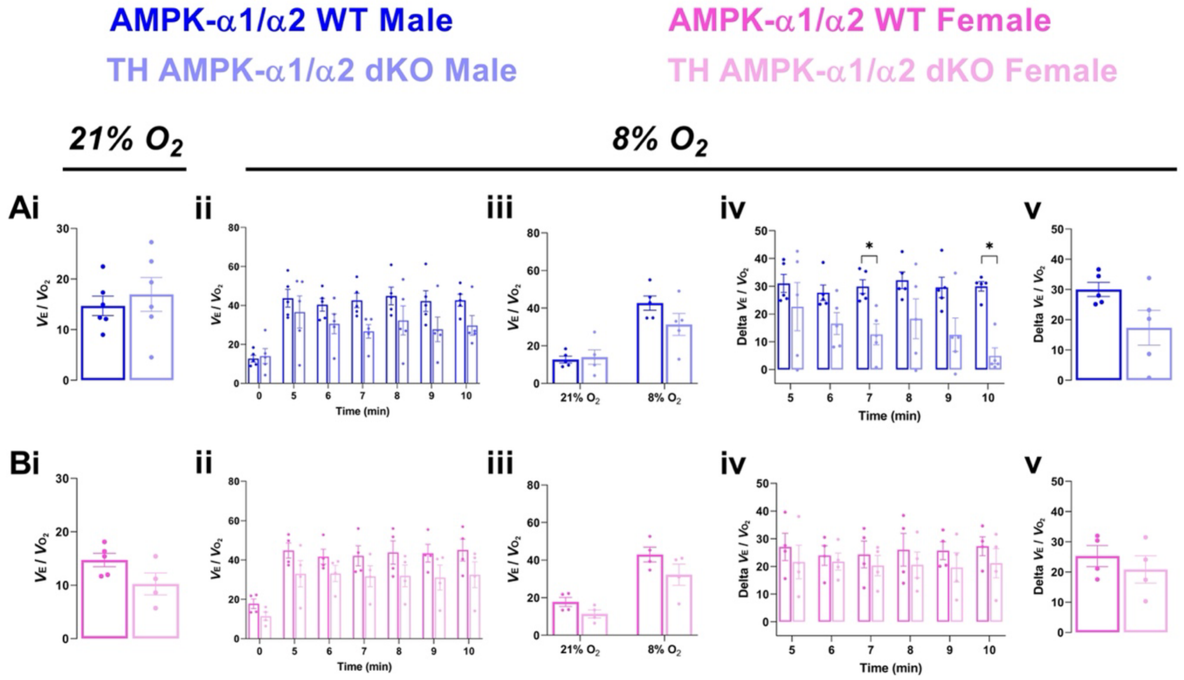

Supplementary Fig. 10: Ventilatory equivalent for oxygen during normoxia and acute exposure to hypoxia in male and female mice. Bar charts and scatter plots show Means ± SEM for ventilatory equivalent for oxygen (V_E/VO_2) in male **(A)** and female **(B)** mice during (**i**) normoxia (21% O_2), (**ii**) before (0) and during minutes 5-10 of 8% O_2, (**iii**) before (21% O_2) and during 8% O_2 (average data for 5-10mins), **(iv)** change in ventilatory equivalent from baseline during minutes 5-10 of 8% O_2, and **(v)** change in ventilatory equivalent from baseline for the average response to 8% O_2 AMPK-α1/α2 wildtype mice (WT; n = 5 males, n = 4-5 females) and in mice with AMPK-α1/α2 deletion in catecholaminergic cells (TH AMPK-α1/α2 dKO; n = 5-6 males; n = 4 females). Significance tested by Student's t-tests (Ai, Bi, Av and Bv) and two-way ANOVA with Sidak post-hoc tests (all other graphs). * = $p<0.05$

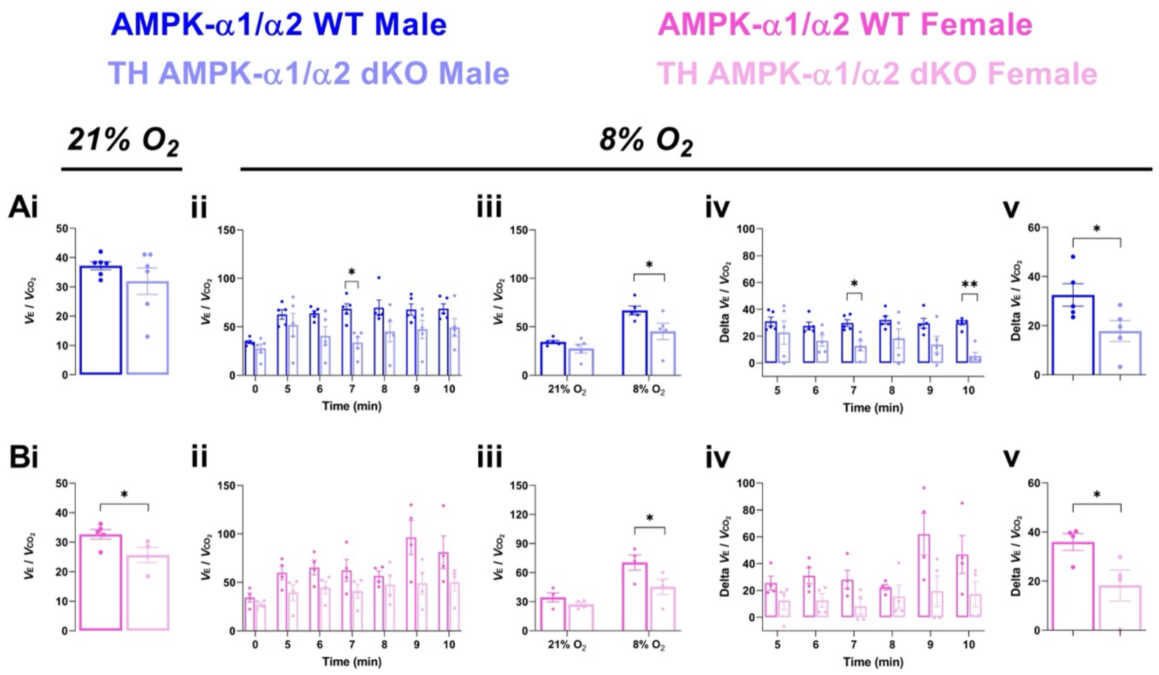

Supplementary Fig. 11: Ventilatory equivalent for carbon dioxide during normoxia and acute exposure to hypoxia in male and female mice. Bar charts and scatter plots show Means ± SEM for ventilatory equivalent for carbon dioxide (V_E/VCO_2) in male **(A)** and female **(B)** mice during (**i**) normoxia (21% O_2), (**ii**) before (0) and during minutes 5-10 of 8% O_2, (**iii**) before (21% O_2) and during 8% O_2 (average data for 5-10min), **(iv)** change in ventilatory equivalent from baseline during minutes 5-10 of 8% O_2, and **(v)** change in ventilatory equivalent from baseline for the average response to 8% O_2 in AMPK-α1/α2 wildtype mice (WT; n = 5 males, n = 4-5 females) and in mice with AMPK-α1/α2 deletion in catecholaminergic cells (TH AMPK-α1/α2 dKO; n = 5-6 males; n = 4 females). * = $p<0.05$. Significance tested by Student's t-tests (Ai, Bi, Av and Bv) and two-way ANOVA with Sidak post-hoc tests (all other graphs).

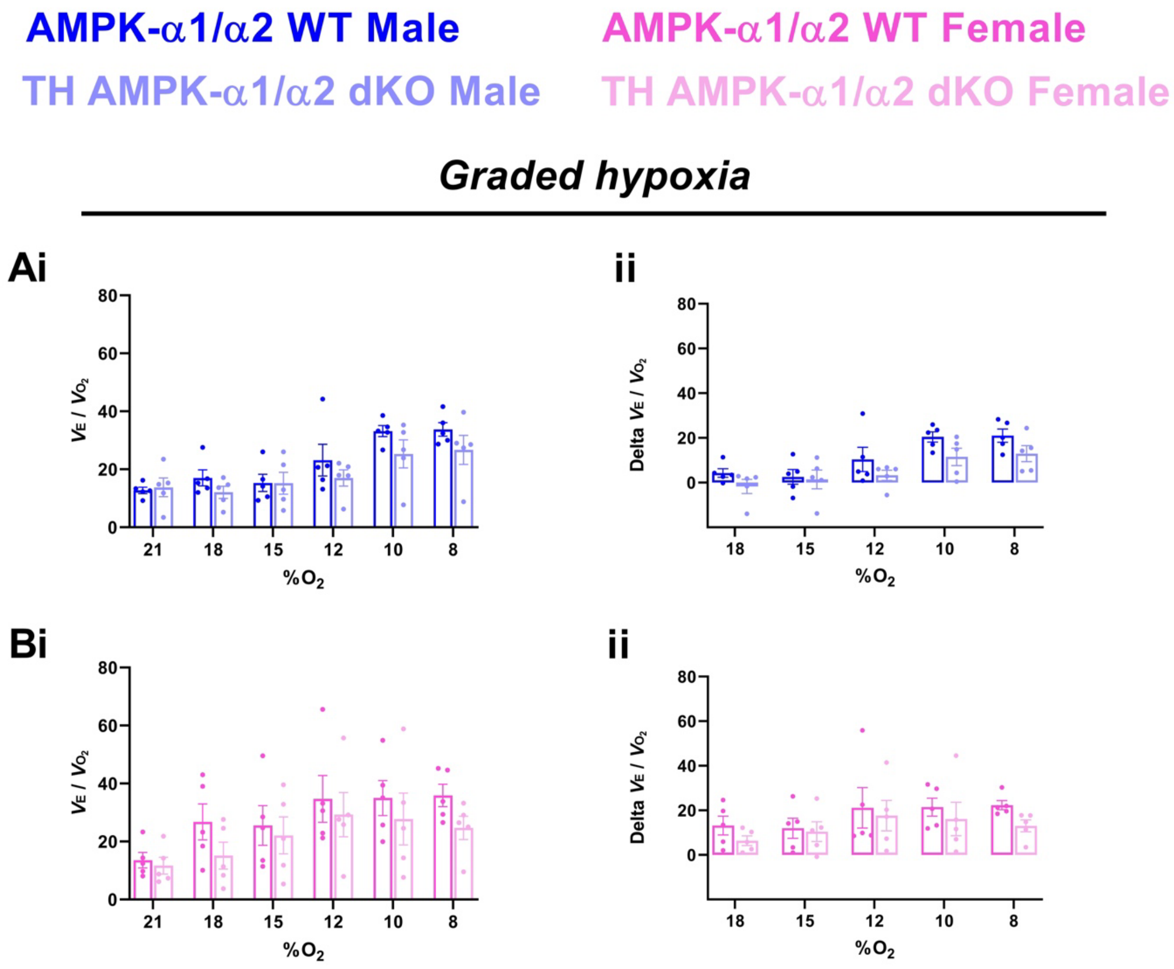

Supplementary Fig. 12: Ventilatory equivalent for oxygen during normoxia and exposure to graded hypoxia in male and female mice. Bar charts and scatter plots show Means ± SEM for ventilatory equivalent for oxygen (V_E/VO_2) in male (**A**) and female **(B)** during (**i**) normoxia (21% O_2; 5 min) and the final minute of a 5 min exposure to 18%, 15%, 12%, 10% and 8% O_2, and (**ii**) change in ventilatory equivalent from baseline in AMPK-α1/α2 wildtype mice (WT; n = 5 males, n = 4-5 females) and in mice with AMPK-α1/α2 deletion in catecholaminergic cells (TH AMPK-α1/α2 dKO; n = 5-6 males; n = 4 females). Significance tested by two-way ANOVA.

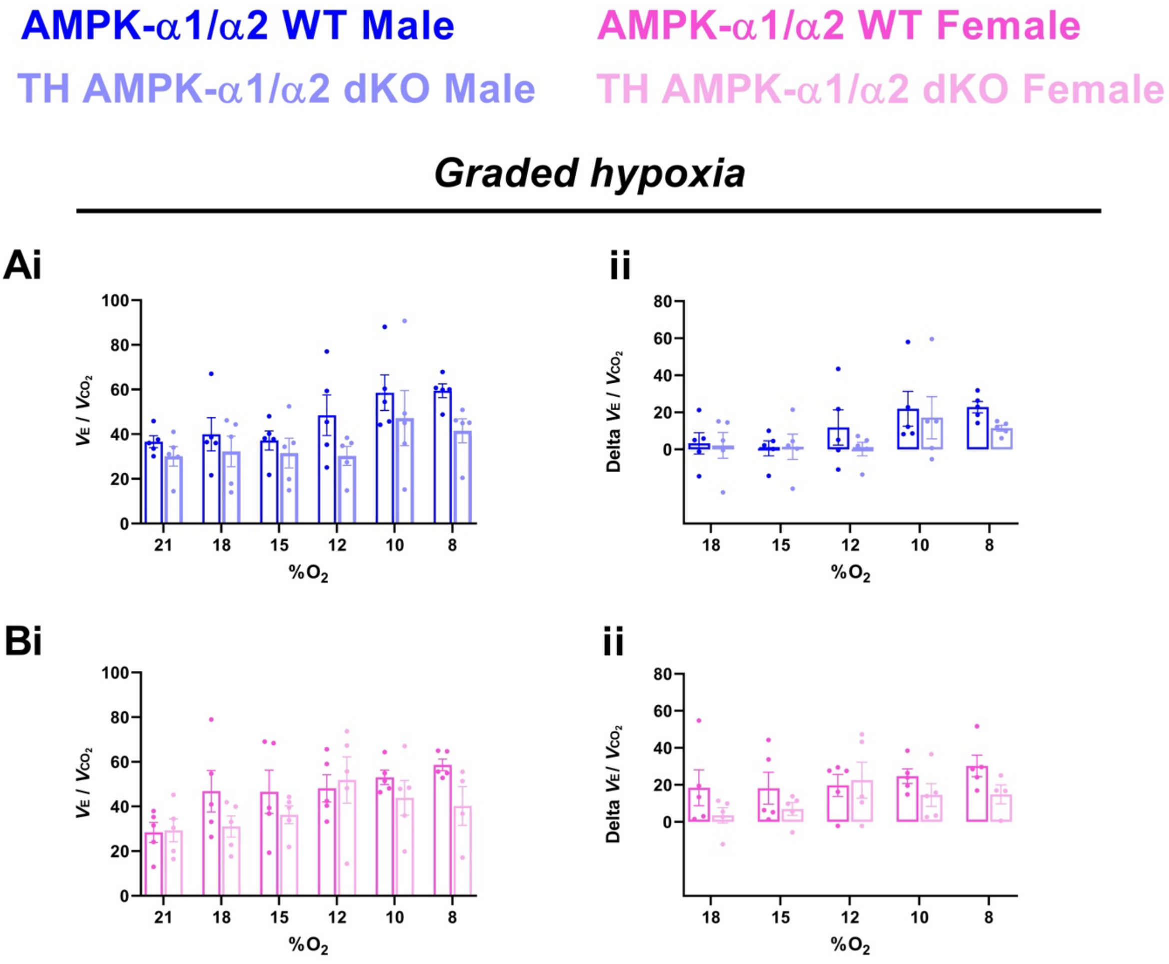

Supplementary Fig. 13: Ventilatory equivalent for carbon dioxide during normoxia and exposure to graded hypoxia in male and female mice. Bar charts and scatter plots show Means ± SEM for ventilatory equivalent for oxygen (V_E/VCO_2) in male (**A**) and female **(B)** during (**i**) normoxia (21% O_2; 5 mins) and the final minute of a 5 min exposure to 18%, 15%, 12%, 10% and 8% O_2, and (**ii**) change in ventilatory equivalent from baseline in AMPK-α1/α2 wildtype mice (WT; n = 5 males, n = 4-5 females) and in mice with AMPK-α1/α2 deletion in catecholaminergic cells (n = 5-6 males; n = 4 females). Significance tested by two-way ANOVA.
